# Characterization of low‐dose ozone‐induced murine acute lung injury

**DOI:** 10.14814/phy2.14463

**Published:** 2020-06-11

**Authors:** Gurpreet Kaur Aulakh, Jessica Andrea Brocos Duda, Claudia Marcela Guerrero Soler, Elisabeth Snead, Jaswant Singh

**Affiliations:** ^1^ Small Animal Clinical Sciences Western College of Veterinary Medicine University of Saskatchewan Saskatoon Canada; ^2^ Universidad Nacional de Colombia Bogota Colombia; ^3^ Veterinary Biomedical Sciences Western College of Veterinary Medicine University of Saskatchewan Saskatoon Canada

## Abstract

Ozone is a toxic and highly reactive gaseous oxidizing chemical with well‐documented adverse health effects in humans. On the basis of animal and human data, environmental guidelines and air quality standards recommend a threshold for exposure of no more than 0.063 ppm of ozone (daily concentrations). This research describes a standardized sensitive model of sterile murine lung inflammation induced by exposing mice to acute (0, 4 or 24 hr), yet low, levels of ozone (0.005, 0.05 or 0.5 ppm), one that are below the current recommendations for what is considered a safe or “ambient” ozone concentration for humans. Ozone led to concentration and time‐dependent phlogistic cell death in the bronchoalveolar lavage, lung epithelial damage and hemorrhage. Interestingly, we observed distinct large bright CD11b positive cells in the bronchoalveolar lavage, upregulation of lung vascular and alveolar ATP synthase as well as plasminogen and bronchiolar angiostatin expression in ozone‐exposed mice, platelet and neutrophil accumulation in the lung vasculature and an eotaxin‐2, IL‐16, CXCL5, CXCL12, and CXCL13 dominant inflammatory response leading to lung injury. Using a fluorescent intravital microscopy set up, we quantified ozone‐induced extensive alveolar cellular damage. We observed ozone‐induced actin filament disorganization, perturbed respiratory mechanics, acute suppression of the alveolar reactive oxygen species (ROS) production and mitochondrial potential in ventilated lungs. We present evidence of systemic, as well as pulmonary toxicity, at 40‐fold lower ozone concentrations than previously reported in mice. The findings are important in establishing a sensitive means of quantifying structural and functional lung disorganization following exposure to an aerosolized pollutant, even at levels of ozone exposure previously thought to be safe in humans.

## Graphical abstract

This study investigates the murine acute pulmonary and systemic inflammatory response to low‐dose ozone exposures. It characterizes lung leukocyte recruitment and acute oxidative alveolar damage in response to low level ozone exposure.

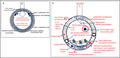

## 1 INTRODUCTION

Ozone (O_3_) is a prevalent environmental pollutant and a major public health concern with hospital admissions and emergency room visits increasing following days of high ambient O_3_ concentrations (Delfino, Murphy‐Moulton, Burnett, Brook, & Becklake, 1997; Stieb, Burnett, Beveridge, & Brook, 1996). Ground level O_3_ is formed by photolysis of reactive intermediates largely created from the burning of fossil fuels (Uysal & Schapira, 2003). O_3_ is responsible for acute respiratory irritation leading to difficulty in breathing (Schnell & Prather, 2017), especially in patients with pre‐existing respiratory disease such as asthmatics and in the elderly population (Dauchet et al., 2018; Fry et al., 2012; Rush, Wiskar, Fruhstorfer, Celi, & Walley, 2018). Even O_3_ concentrations below the recommended 8 hr standards of 0.063 ppm (Vanos, Cakmak, Kalkstein, & Yagouti, 2015) are sufficient to reduce lung function in asthmatic children. According to the latest report issued by the Canadian Census Health and Environment Cohort (CanCHEC), Saskatchewan or the Eastern Prairie region is amongst the highest risk zones for O_3_ exposure and particulate matter related lung cancer, ischemic heart disease and COPD (Cakmak et al., 2018). Thus, it is imperative to understand the biological effects of O_3_ at concentrations feasible in the environment.

Acute lung injury (ALI) often results from excessive lung damage that leads to alveolar barrier disruption with secondary lung edema and progressive loss of lung function. Bacterial pneumonia or endotoxin (or lipopolysaccharide, LPS) exposure induce immediate (i.e., within 1 hr) release of IL‐1β dependent cytokines and chemokines and lead to enzymatic cleavage of plasminogen/plasmin into angiostatin fragments (Falcone, Khan, Layne, & Fernandes, 1998; Hamacher et al., 2002). Innate immune chemokines such as tumor necrosis factor α (TNFα), interleukin‐1β (IL‐1β), keratinocyte chemoattractant (KC), and macrophage inflammatory protein (MIP1α) lead to increased expression of adhesion molecules like platelet endothelial cell adhesion molecule‐1 (PECAM‐1 or CD‐31), P‐selectin (CD‐62P), and CD11b (integrin α_M_). These molecules mediate platelet, neutrophil, and mononuclear infiltration into the lungs (Berman & Muller, 1950; Grommes et al., 2012; Schenkel, Chew, Chlipala, Harbord, & Muller, 2006). Animal models have also helped characterize potential mechanisms of pulmonary (Dowell, Lohrbauer, Hurst, & Lee, 1970; Michaudel, Fauconnier, Jule, & Ryffel, 2018) Bhattacharya & Westphalen, 2016) as well as multiple organ injury (Bouthillier et al., 1998; Erickson et al., 2017; Henriquez et al., 2018; Kasahara et al., 2014; Thomson, Pilon, Guenette, Williams, & Holloway, 2018) resulting from O_3_ exposure. These studies show that a 3–6 hr exposure to 0.8 or 2 ppm O_3_ induces recruitment and accumulation of lung monocytes and neutrophils at 24 hr (Francis et al., 2017; Tighe et al., 1950). However, there is paucity of data on the early (within 1 hr) lung cellular profiles in response to O_3_ exposure. To this end, we ascertained the immediate alveolar as well as vascular (pulmonary and systemic) inflammatory cell recruitment and lung chemokine gradients in response to O_3_ exposure in concentrations upto 0.05 ppm. We hypothesized that a low level of O_3_ is capable of inducing the hallmarks of lung injury, namely CD11b‐dependent Gr1 leukocyte recruitment, lung edema, histological evidence of lung damage, chemokine release and enhanced expression of plasminogen and angiostatin fragments and adhesions molecules (CD‐31, CD‐62P).

As O_3_ potently induces instant cell death, we asked the question if 2 hr of continuous exposure to 0.05 ppm O_3_ concentration will cause enough damage to the alveolar cytoskeleton to induce an adaptive response, which is accompanied by mitochondrial activation leading to upregulation of cell survival proteins such as ATP synthase (ATPβ) and signaling molecules such as reactive oxygen species (ROS). We, thus, performed intravital lung imaging to assess O_3_‐induced damage to the alveolar actin cytoskeletal arrangement, and the effect on mitochondrial ROS generation and mitochondrial potential. In addition, we stained lung sections for regional lung ATPβ expression and quantification.

## 2 MATERIALS AND METHODS

### 2.1 Mice

The study design was approved by the University of Saskatchewan's Animal Research Ethics Board and adhered to the Canadian Council on Animal Care guidelines for humane animal use. Six‐eight week old male C57BL/6NJ (Stock No. 005304) mice were procured from Jackson Labs.

### 2.2 Reagents and chemicals

All chemicals utilized for the experiments were purchased from Sigma‐Aldrich Chemicals unless otherwise indicated. Intravenous dyes such as Alexa 555 tagged phalloidin (Life Technologies Inc.) was superfused to highlight intracellular actin dynamics. Intracellular reactive oxygen species (ROS) were detected by the IMAGE‐iT LIVE Green ROS Detection Kit which consists of 5‐(and‐6)‐carboxy‐2',7'‐dichlorodihydrofluorescein diacetate (carboxy‐H2DCFDA, Life Technologies Inc.). To determine intracellular mitochondrial activation, mitochondrial redox potential was imaged using reduced mitotracker orange (Life Technologies Inc.).

### 2.3 Ozone exposures

For O_3_ exposures, mice were continuously exposed in an induction box for the desired times. These mice had free access to food and water while housed in the custom induction box. O_3_ (±0.02 ppm) was generated, at 3 L/minute, from ultra‐high‐purity air using a silent‐arc discharge O_3_ calibrator cum generator (2B Technologies). Constant chamber air temperature (72 ± 3°F) and relative humidity (50 ± 15%) were maintained. O_3_ concentrations were measured using a real‐time O_3_ monitor (2B Technologies).

### 2.4 Experimental design

#### 2.4.1 Ozone time and dose titration

**FIGURE 1:**
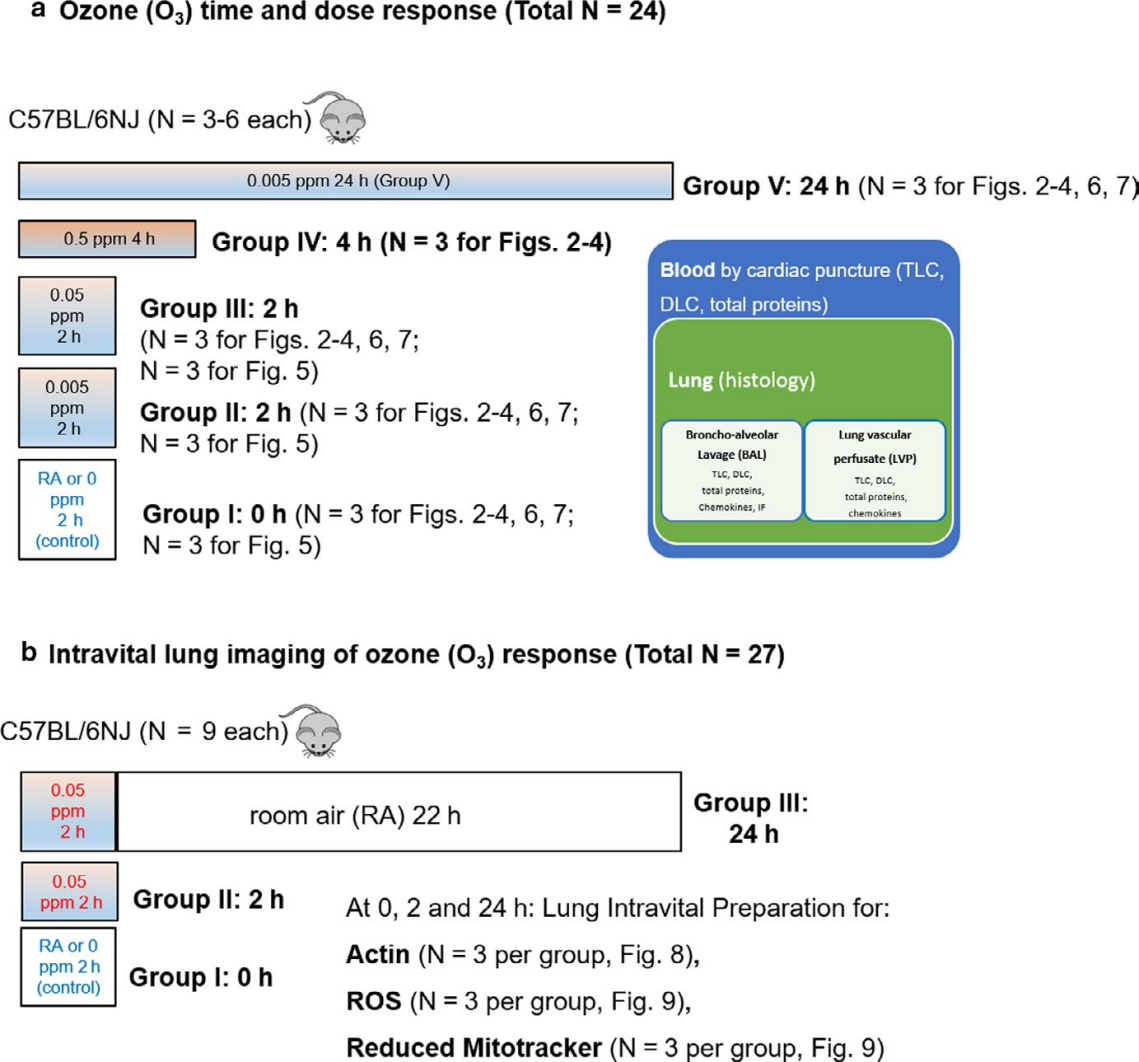
Experiment design. (a) Experiment design for room air (RA) (i.e.,), 0 hr or control (Group I, *N* = 6), ozone (O_3_) at 0.005 ppm for 2 hr (Group II, *N* = 6), 0.05 ppm for 2 hr (Group III, *N* = 6), 0.5 ppm for 4 hr (Group IV, *N* = 3), or 0.005 ppm for 24 hr (Group V, *N* = 3 per group) in the C57BL/6NJ sub‐strains (black mice). Schematic of cellular compartments assayed from the lungs (Bronchoalveolar lavage/BAL, lung vascular perfusate/LVP) and peripheral/cardiac blood of each mouse. BAL and LVP were assayed for Total leukocyte counts/TLC, differential leukocyte counts/DLC, total proteins, chemokines and BAL CD11b and Gr1 localization. Left lungs were stained with H&E for histology. (b) Experimental design for intravital lung imaging in control or room air (RA) 0 hr (Group I) and ozone (O_3_) (0.05 ppm for 2 hr) imaged at 2 (Group II) or 24 (Group III) h. *N* = 9 per group: From each group, 3 mice each were imaged for actin (Rhodamine phalloidin, 1:1,000 dilution), reactive oxygen species (ROS) (5‐(and‐6)‐carboxy‐2',7'‐dichlorodihydrofluorescein diacetate (carboxy‐H_2_DCFDA), 1:1,000 dilution), and mitotracker potential (Reduced mitotracker, 1:1,000 dilution)

Six control mice were exposed to room air (RA) in a custom induction chamber for 2 hr (designated as group I or 0 hr). In groups II and III, six mice were continuously exposed for a short (2h) duration at 0.005 ppm and 0.05 ppm O_3,_ respectively. In group IV, three mice were continuously exposed for a longer (4 hr) duration and at a higher concentration of 0.5 ppm O_3_. In group V, three mice were continuously exposed for the longest (24 hr) duration but to the lowest (0.005 ppm) O_3_ concentration to investigate the effects of prolonged O_3_ exposure at concentrations that are considered safe for humans. Immediately after respective O_3_ exposures (i.e., 0, 2, 4, or 24 hr), mice were anesthetized intraperitoneally with ketamine (200 mg/kg) and xylazine (10 mg/kg) and prepared for further maneuvers. The experimental design is summarized in Figure 1a. Peripheral blood was collected by cardiac puncture. Mice were then tracheostomized, a custom noncollapsible polyethylene cannula was placed just before the end of tracheal bifurcation. Broncho‐alveolar lavage (BAL) was collected with three consecutive washes, each with 0.5 ml PBS (phosphate buffered saline). The descending thoracic aorta was snipped at the mid‐thoracic region. After clearing residual blood, lung vascular perfusate (LVP) was collected by perfusion through the right ventricle with PBS (0.25 ml × 2). The right lung lobes were tied, resected, and flash frozen in liquid nitrogen and then stored at −80°C for further analysis. The left lung was perfused with 0.5 ml freshly prepared 4% paraformaldehyde from a 20 cm water column to enable in situ fixation for 10 min and later cryo‐embedded. The collected samples are shown in the schematic in Figure 1a. Thus, for groups I‐III (3 × 3 = 9 mice), three additional mice were added in order to perform BAL immune‐fluorescence (Gr1 and CD11b) staining, apart from three mice per group (groups I‐V, 3 × 5 = 15 mice) used for bright‐field cytology and protein quantifications from BAL, vascular perfusate and blood samples, and lung histology.

#### 2.4.2 Intravital imaging of ozone lung exposure

The objective of this experiment was to compare O_3_‐induced dynamic changes in lung architecture (actin), mitochondrial activity, and ROS production by intravital microscopy to those of control mice exposed to room air (RA). Nine control mice were exposed to RA in a custom induction chamber for 2 hr. Immediately after RA exposure, the mice were prepared for intravital lung microscopy. These mice are designated as Group I or 0 hr time‐point. For Group II (or 2 hr time‐point), nine mice were exposed to 0.05 ppm O_3_ for 2 hr. Immediately after O_3_ exposure (that is at 2 hr time‐point), mice were prepared for intravital lung microscopy. For Group III (or 24 hr time‐point), nine mice were exposed to 0.05 ppm O_3_ for 2 hr and recovered for 22 hr in a normal RA cage, after which the mice were prepared for intravital lung microscopy (that is at 24 hr time‐point). For each of these three groups, lungs from three mice were superfused with Alexa 555‐phalloidin for actin visualization, three mice with the ROS dye (carboxy‐H2DCFDA) for ROS imaging, and three mice for reduced mitotracker orange for active cellular mitochondria visualization. The experimental design for the live imaging experiments is summarized in Figure 1b.

### 2.5 End‐points

We evaluated end‐points “a through f” for the O_3_ time and dose titration study and the endpoint “g” for the intravital lung imaging study.

#### 2.5.1 Total (TLC) and differential (DLC) leukocyte counts

Blood, BAL and lung vascular perfusate samples were centrifuged for 10 min at 3,000 rpm. The supernatants were flash frozen and stored at −80°C until further analysis. The cells were reconstituted in PBS. TLC was performed by counting BAL, blood, or lung perfusate cells on a hemocytometer. Trypan blue dye exclusion was utilized to quantify live and dead BAL cells under light microscopy. Blood TLC is presented as x10^6^ cells per mL. BAL and lung perfusate TLC are expressed in ×10^3^ and ×10^6^cells per collection, respectively. Acetic acid (2%) was added to lyse RBCs, in a 1:10 ratio for blood TLC and 1:2 ratio for lung vascular perfusate TLC. The cells (not more than 1 × 10^6^ per slide) were then centrifuged (Shandon cytospin, Thermoscientific) to prepare cyotspins and stained with Diffquick for DLCs. A minimum of 200 cells were counted for differential leukocyte cell counts (DLCs) or debris. Debris was identified as incomplete or damaged cellular fragments, extra‐cellular nuclear material or condensed cells. A clump of such material was accounted as a count of one when quantifying debris.

#### 2.5.2 BAL cytospin CD11b and Gr1 staining

BAL cytospin samples from groups I, II, and III were stained for the CD11b and Gr1 surface markers (2 µg anti‐CD11b PE, and 2 µg anti‐Gr1 Alexa 488, respectively). Nuclei were stained with DAPI (4′,6‐diamidino‐2‐phenylindole). Cytospin preparations were also stained with rat IgG2b kappa isotype control antibody to validate the staining protocol. Images were acquired under a wide‐field upright microscope equipped with a Sony camera.

#### 2.5.3 Lung immune‐fluorescent staining

Lung cryosections from groups I‐IV were stained with two different primary antibody combinations. Combination 1 was comprised of direct immune‐fluorescent staining with Gr1‐488 and CD11b‐PE‐labeled antibodies. The rest of the combinations were comprised of indirect immunofluorescent staining protocols. Combination 2 was comprised of either mouse ATPβ and rabbit plasminogen antibodies or mouse ATPβ and rabbit CD31 (to visualize ATPβ localization with respect to endothelial cells). Combinations 3 and 4 were stained from the short RA or O_3_ exposure groups I and III to ascertain the immediate changes in lung plasminogen, angiostatin, CD62P, and vwf protein fluorescence. Combination 3 comprised of rabbit vwf and rat Gr1 antibodies. Combination 4 comprised of goat CD62P (P‐selectin) and rabbit angiostatin antibodies. The respective secondary antibody combinations were anti‐mouse Alexa 488 and anti‐rabbit Alexa 568 for combination 2, anti‐rabbit Alexa 488 and anti‐rat Alexa 568 for combination 3, and anti‐goat Alexa 488 and anti‐rabbit Alexa 568 for combination 4.

Briefly, all sections were hydrated in tris buffered saline (TBS). Before overnight incubation with primary antibodies at 4°C, sections were permeabilized with 0.1% triton X‐100 for 5 min, followed by washes in TBS buffer. Nonspecific binding was blocked with 5% bovine serum albumin for 30 min at room temperature. For combination 2, which comprised of ATPβ antibody raised in mouse, an additional 20 min room temperature blocking step was introduced with Fc blocking buffer. Following overnight primary antibody incubation in a humidified chamber at 4°C and TBS washes to remove the unbound primary antibody, secondary antibody was then incubated for 30 min at room temperature. DAPI was incubated during the last 10 min before the secondary antibody wash. Sections were cover slipped with ProlongGold™ anti‐fade mounting media and sealed after overnight drying in the dark at room temperature.

#### 2.5.4 BAL, lung vascular perfusate, and blood protein analysis

To quantify O_3_‐induced perturbations of the vascular barrier or the relative oncotic pressure in the various compartments, that is, the alveolar (interstitial), lung vascular perfusate (vascular), and peripheral blood (systemic) compartments, we measured total protein content in the collected fluids. Supernatant fractions were analyzed for their total protein concentration using a standard colorimetric assay (Pierce 660 nm protein assay, Thermoscientific).

#### 2.5.5 BAL, lung vascular perfusate, and bone marrow chemokine analysis

BAL supernantants, from groups I‐V, were analyzed for chemokines using a 33‐plex magnetic bead‐based immunoassay (Bio‐rad Laboratories Ltd.). To understand the chemokine gradients established by 24 hr of continuous 0.005 ppm O_3_ exposure (group V), supernatants of lung vascular perfusate and bone marrow samples were also assayed. This group was chosen in order to assess the establishment of chemokine gradients in the different compartments after 24 hr of continuous O_3_ exposure, in addition to avoiding prohibitive cost of 33‐plex assays. The following chemokines were analyzed: CXCL13 (B‐lymphocyte chemoattractant), CCL27 (IL‐11 R‐alpha‐locus chemokine (ILC)), CXCL5 (epithelial‐derived neutrophil‐activating peptide 78 (ENA‐78)), CCL‐11 (eotaxin‐1), eotaxin‐2 (CCL‐24), CX3CL1 (fractalkine), GM‐CSF (CSF‐2), CCL1, IFNγ (interferon gamma), IL‐10 (interleukin‐10), IL‐16 (interleukin‐16), IL‐1β (interleukin‐1 beta), IL‐2 (interleukin‐2), IL‐4 (interleukin‐4), IL‐6 (interleukin‐6), CXCL‐10 (interferon gamma‐induced protein 10 (IP‐10)), CXCL11 (Interferon‐gamma‐inducible protein 9 (IP‐9)), KC (keratinocyte chemoattractant), MCP‐1 (monocyte chemoattractant protein‐1), MCP‐3 (monocyte chemoattractant protein‐3), MCP‐5 (monocyte chemoattractant protein‐5), MDC (macrophage‐derived chemokine (CCL22)), MIP‐1α (macrophage inflammatory protein‐1 alpha), MIP‐1β (macrophage inflammatory protein‐1 beta), MIP‐2 (macrophage inflammatory protein‐2), MIP‐3α (macrophage inflammatory protein‐3 alpha), MIP‐3β (macrophage inflammatory protein‐3 beta), RANTES (regulated on activation, normal T cell expressed and secreted (CCL5)), CXCL‐16, CXCL‐12/SDF‐1alpha (stromal cell‐derived factor 1), TARC (thymus and activation regulated chemokine (TARC)), TECK (Thymus‐Expressed Chemokine (CCL25)), and TNFα (tumor necrosis factor alpha).

#### 2.5.6 Hematoxylin and eosin (H&E) histology

H&E staining was performed on cryoembedded lung sections for all groups.

#### 2.5.7 Lung intravital microscopy

Briefly, anesthetized and intubated mice were ventilated with positive end expiratory pressure (PEEP) settings of 2 cm H_2_O pressure, a respiratory rate of 100–120 breaths per minute and a tidal volume of 200 µl. Lungs were exposed by carefully cutting the ribs from the left latero‐dorsal side after which the lungs were gently suctioned under 25–30 mm Hg vacuum to a custom‐made optically transparent thoracic window (Looney et al., 2011). An Olympus BX51W1 fixed‐stage Gibraltar upright microscope was upgraded to laser powered sources of 488 and 561 nm excitation wavelengths (Coherent Light Source), for performing intravital microscopy. The lasers were coupled through a liquid light guide. A DAPI/GFP/TRITC triple bandpass dichroic excitation emission filter, corresponding to 405/488/561 nm, was mounted on an Olympus BX2 cube. The transmitted visible light was collected by a consumer‐grade Canon HV20A CMOS RGB camera. These signals were displayed and recorded on a computer monitor utilizing a video capture card. The fluorescent emission was collected using a scientific grade ICCD XR/Mega 10 Extreme camera (Stanford Photonics, Inc.). The effective pixel size of the ICCD camera was 10 µm with a image capture rate of 30 fps. To minimize laser toxicity for the two‐light fluorescent signal acquisition, a camera trigger with a frame rate of 15 fps for the first laser excitation was followed by a 2‐s pause and then captured with the second laser excitation at 15 fps. The signals were displayed and recorded onto a computer monitor via image acquisition and control software (*PiperControl*; Stanford Photonics Inc.). Videos were acquired under 10× air or 20× water objectives with numerical apertures of 0.30 and 0.95, respectively. Videos were acquired for a maximum of 30 min to avoid laser damage to tissue. This time was considered adequate without inducing any significant laser‐induced tissue damage as assessed by changes in the average baseline ROS concentration and reduced mitotracker intensity over time.

### 2.6 Image analysis

Collected image data (.tiff) files were processed and analyzed in Fiji ImageJ (https://imagej.net/Fiji/Downloads). For Diff‐Quick stained BAL, lung perfusate, and blood cytospins, the oil objective images were inverted and adjusted for enhanced local contrast to visualize damaged cells, extracellular nuclear material, platelets and platelet clumps. A total of 200 cells were counted manually to calculate the DLC.

For CD11b and Gr1 stained BAL cytospins, between 50 and 100 cells were manually thresholded and the corresponding regions of interest were selected in the CD11b and Gr1 channels. A log fold drop in fluorescent intensity was considered as the negative population of the corresponding fluorescent signal. Percentage of positive or negative cells were calculated and reported. For the positive cells, the integrated fluorescent pixel intensities and pixel frequency distributions (histograms) were objectively analyzed for CD11b and Gr1 expression by each cell. Cellular fluorescent intensity distributions (integrated fluorescence intensity, maxima, standard deviations in the fluorescence intensities, maximum fluorescence intensity) as well as pixel frequency and spatial distributions (skewness and kurtosis, respectively) were plotted for Alexa 488 (Gr1) and the PE (CD11b) channels. Cell perimeters were also plotted to evaluate any change in cell size after O_3_ exposure. The average intra‐assay and inter‐assay coefficient of variation (CV) for integrated fluorescence intensity of Gr1 in control group are 0.502 and 0.137, respectively. The average intra‐assay and inter‐assay CV for integrated fluorescence intensity of CD11b in control group are 0.503 and 0.116, respectively.

For lung immune‐fluorescent quantification, bronchiolar, vascular (arteriolar and venular) as well as alveolar septal (alveolar) regions were manually thresholded, and perimeter normalized fluorescence intensity was analyzed.

The actin, ROS, and mitotracker image sequences were registered using the rigid registration plugin in Fiji. The 16‐bit images were only corrected for brightness and contrast and not thresholded. Features such as air‐spaces were selected using the feature's pixel threshold and analyzed over multiple frames (100–200) to detect respiratory motion. For ROS and mitotracker image sequences, each sequence was compared on a common scale (to cover the entire histogram range) and visualized on a color scale. Motion analysis of alveolar area, ROS or mitotracker positive lung parenchyma in a sub‐pleural field of view was executed after thresholding the air‐spaces from stained area, converting the desired sequence into binary images and then computing the percentage of area covered by the desired read‐out (i.e., alveolar space, ROS, or mitotracker positive) out of the full field of view.

For ROS and reduced mitotracker image analysis, the difference images (T1‐T2, that is, start and end images for 10 min of acquisition) were an inverse of the difference image is displayed for visualization of the respective activities. The (average and maximum) fluorescent intensity gray values are reported from the original 16‐bit images obtained from ROS, reduced mitotracker or actin stained sub‐pleural alveoli. The image sequences were converted and saved as*.avi* (for the ICCD camera) or*.mp4* (for the Canon camera) movie files.

### 2.7 Statistical analysis

The results are expressed as mean ± *SEM*. A minimum of three mice were used per group. Two‐way ANOVA was used to identify the effect of 2 factors, cell type (eosinophils, neutrophils, mononuclear cells, platelets, platelet clumps, debris, and total cell counts) and ozone exposure (0 2 hr, 0.005 2 hr, 0.05 2 hr, 0.5 4 hr, 0.005 24 hr), after homoscedasticity testing. Bonferroni's multiple comparisons (which corrects for the variance from multiple factors (7 cell types × 5 exposure groups) as well as adjusts the *p*‐values) were then applied for analyzing effects between groups for each cell‐type. For chemokine data analysis, one‐way ANOVA p‐values were adjusted for false discovery rate according to the Benjamini and Hoshberg correction. Pulmonary capillary diameters were analyzed by Mann–Whitney U test as data were not normally distributed. The alveolar perimeter and circularity were analyzed by unpaired two‐tailed *t* test. For rest of the imaging experiments, we analyzed the results using a one‐way or two‐way ANOVA followed by Sidak's multiple comparisons, depending on the number of derived parameters in analysis. *p*‐values < .05 were considered significant. All data analysis was performed on Graphpad Prism (v8.3.0) software.

## 3 RESULTS

### 3.1 Low O_3_ levels induce marked alveolar cell death, and platelet and neutrophil pooling in the LVP

**FIGURE 2:**
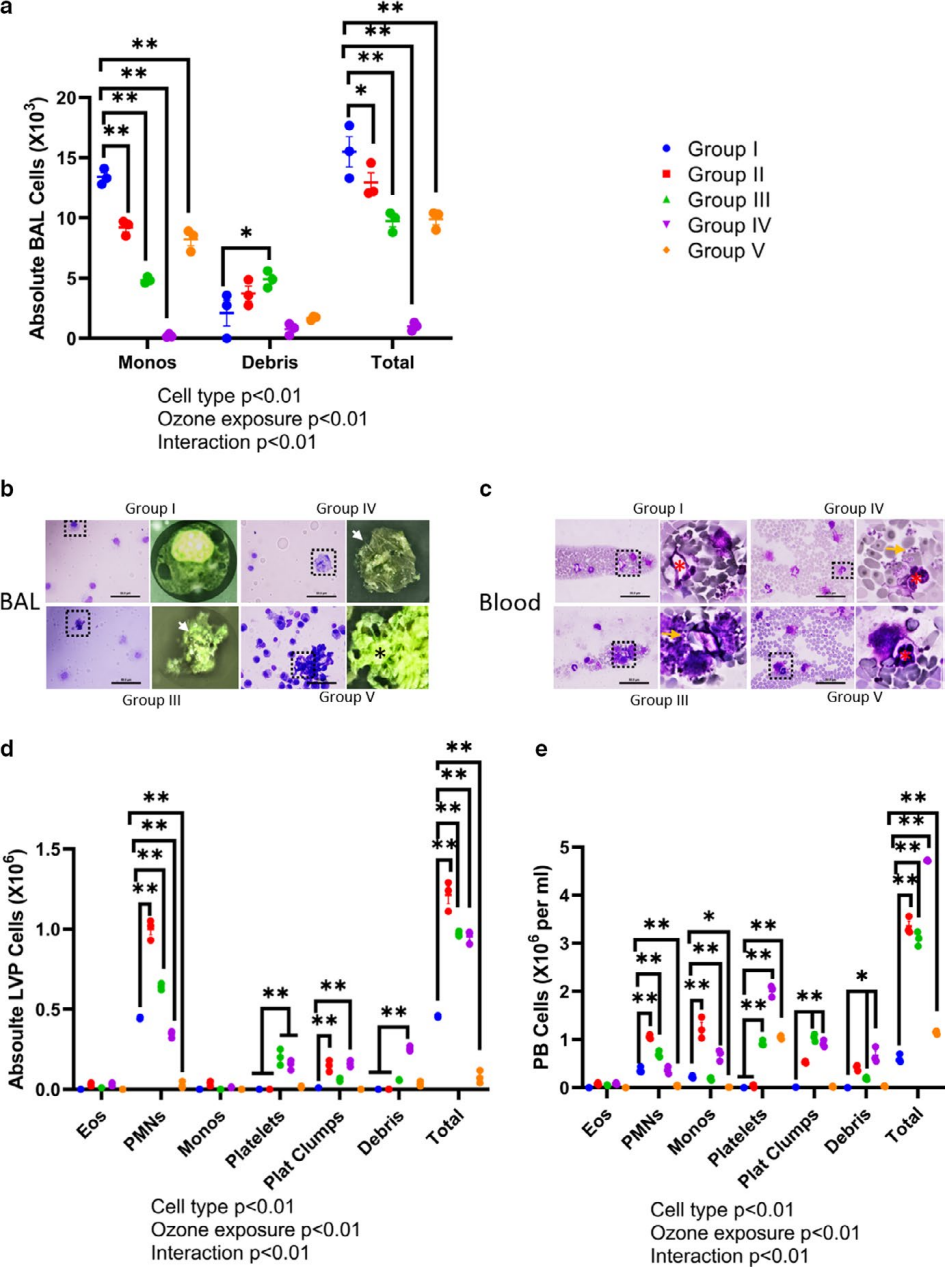
Cellular analysis. (a) Differential and total bronchoalveolar lavage (BAL) cellular analysis in Groups I‐V. (b) BAL and (c) blood cytospins from mice exposed to room air or control (RA i.e. 0 hr or Group I), 0.05 ppm for 2 hr (Group III), 0.5 ppm for 4 hr (Group IV), or 0.005 ppm for 24 hr (Group V). BAL cells showing cytoplasmic aggregates and condensed nuclear material are shown in the magnified views marked by white arrows. Extracellular chromatin networks are marked by asterisks in the magnified view of group IV BAL. Peripheral blood cells are magnified to show neutrophils in red asterisks (*). Group of platelets is marked with yellow arrows. (d) Differential and total lung vascular perfusate (LVP) cell counts. The 2 hr 0.05 ppm O_3_ exposure caused significant increase in the total cell counts compared to group I (*p* < .01). (e) Differential and total peripheral blood (PB) cell counts. The 2 hr 0.05 ppm O_3_ exposure caused significant increase in the total cell counts compared to group I (*p* < .01). * *p* < .05, ** *p* < .01 represent pair‐wise statistical significance

At baseline (Group I, time 0 hr), >90% of cells in the BAL were mononuclear. Exposure to 0.005 (Group II) and 0.05 ppm O_3_ (Group III) for 2 hr induced an 8.3% (*p* = .0427) and 34.6% (*p* < .01) reduction, respectively, in the recovered BAL cells, when compared to control or 0 ppm O_3_ (Group I) (Figure 2a). A further increase in the O_3_ dose to 0.5 ppm and exposure time to 4 hr (Group IV) reduced BAL cell recovery by 93.6% of control. Longer (24 hr) exposure to 0.005 ppm O_3_ (Group V) also suppressed the BAL cell recovery (Figure 2a) by 36.1% of control. BAL cells recovered after O_3_ exposures were either mononuclear or dead cells (Figure 2a,b). Although not shown, we confirmed this finding with viable cell counts following trypan blue dye exclusion. Longer (24 hr) O_3_ exposure induced accumulation of phlogistic debris in the BAL, comprised of extracellular nuclear material and dead cells (Figure 2b).

Along with the decrease in BAL cell counts, O_3_‐induced significant increase in lung vascular perfusate and peripheral blood TLC (*p* < .01, Figure 2d,e). In addition, exposure to increasing concentrations of O_3_ also led to a dramatic change in the nature of the cells seen in the vascular perfusate. Short (2 hr) 0.05 ppm O_3_ exposures (Group III) induced significant accumulation of the neutrophils (20%–40%, *p* < .01), platelets (>30%, *p* < .01) as well as platelet clumps (*p* < .01) in the lung vascular perfusate (Figure 2d) and peripheral blood (Figure 2c,e). Longer O_3_ exposures (0.5 ppm 4 hr or 0.005 ppm 24 hr) led to a significant increase in the platelets (>40%–90% of the total cells) in lung vascular perfusate (Figure 2d) and peripheral blood (Figure 2c,e).

The suppression in neutrophil counts, accumulation of chromatin cellular debris and platelet clumps in the vascular compartments indicate extensive cell death, dysregulated hemostasis, and platelet activation after prolonged O_3_ exposures (4 and 24 hr).

### 3.2 Low levels of O_3_ affects total protein content in BAL, LVP, and peripheral blood

**FIGURE 3:**
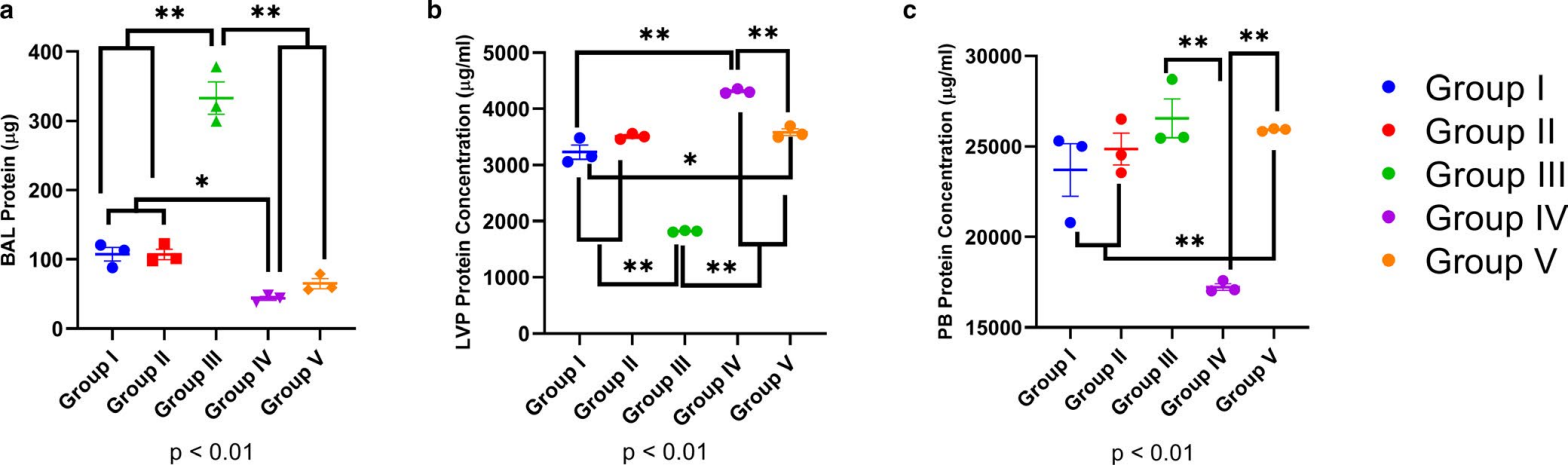
Ozone (O_3_)‐induced protein extravasation and alterations. (a) Bronchoalveolar lavage (BAL) total protein content in Groups I‐V. (b) Lung vascular perfusate (LVP) total protein content in Groups I‐V. (c) Peripheral blood (PB) total protein content in Groups I‐V. * *p* < .05, ** *p* < .01 represent pair‐wise statistical significance

Exposure to 0.05 ppm O_3_ for 2 hr (Group III) induced a 3.23‐fold increase in the BAL protein (Figure 3a). Longer exposures of 4 (Group IV) or 24 hr (Group V) produced a net reduction in BAL protein content (Figure 3a) indicating hypo‐osmotic alveolar fluid. The 0.05 ppm 2 hr O_3_ exposure reduced lung vascular perfusate protein (*p* < .01, Figure 3b) again indicating hypo‐osmotic lung vascular compartment. Longer exposures to O_3_ (Group IV and V), instead, increased the protein in lung vascular perfusate (Figure 3b). Although the longer O_3_ exposures reduced total proteins in BAL compared to the lung vascular perfusate, these numbers correlated with the total cell counts in both the compartments (Figure 2a,b, Figure 3a,b).

The shorter (2 hr) 0.05 ppm or long (24 hr) 0.005 ppm O_3_ exposures did not affect peripheral blood protein content compared to group I (0 ppm) but O_3_ exposures of 0.5 ppm for 4 hr (Group IV) reduced total protein in peripheral blood (*p* < .01, Figure 3c) indicating hypo‐osmotic peripheral blood.

### 3.3 Low levels of O_3_ induce lung hemorrhage and bronchiolar damage

**FIGURE 4:**
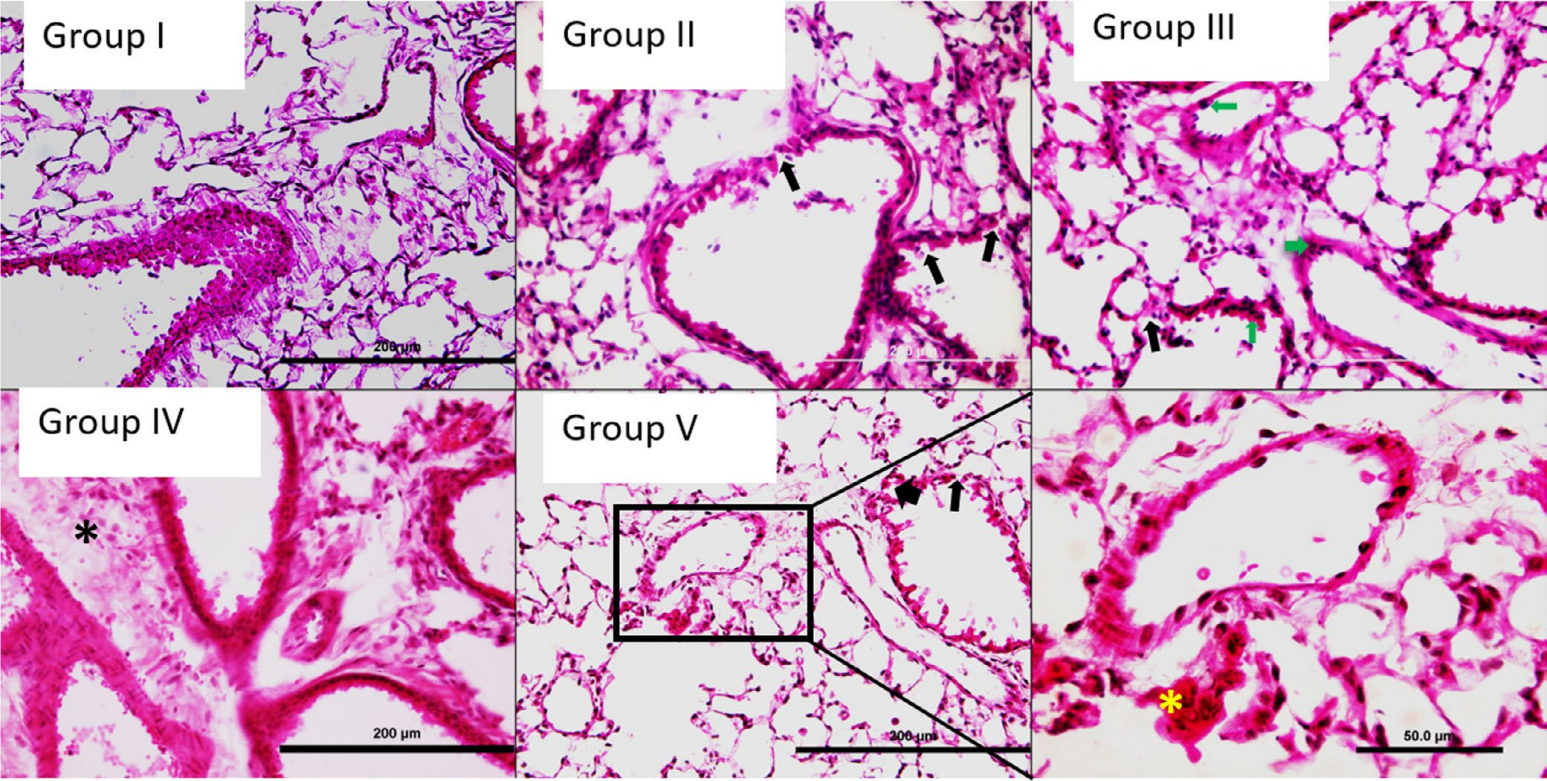
Hematoxylin and eosin (H&E) histology. Ozone (O_3_) induced lung cryosection H&E histology in Groups I‐V. Regions of alveolar epithelial damage are marked by solid black arrows. Neutrophils are marked with green arrows. Black asterisks (*) represent the hemorrhagic region in group IV lung sections and yellow asterisks (*) represent the fibrotic regions in the inset from group V

Short (2 hr) O_3_ exposures of 0.005 ppm (group II) or 0.05 ppm (group III) induced signs of alveolar, peri‐vascular, and peri‐bronchiolar hemorrhage in lung tissues compared to group I (Figure 4. Endothelial and bronchiolar cell damage was marked in groups IV and V (Figure 4a), indicating dose and time‐dependent progression of lung damage. Notably, group IV showed frank hemorrhage and group V showed fibrin clusters within alveoli. O_3,_ at 0.05 ppm for 2 hr, also induced coagulative inflammatory changes in multiple organs (data not shown).

### 3.4 Low levels of O_3_ induce recruitment of distinct CD11b positive as well as negative mononuclear cells

**FIGURE 5:**
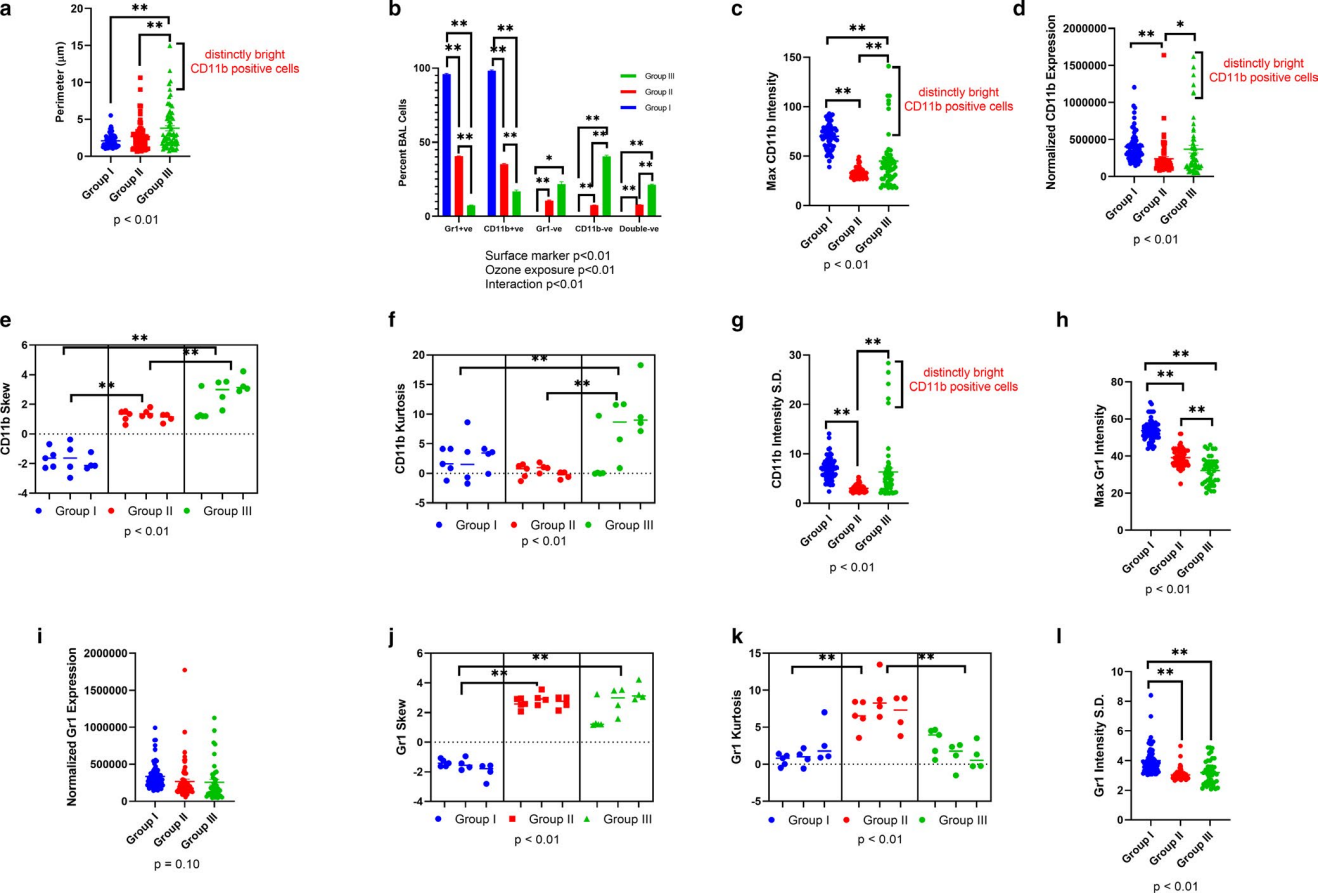
Bronchoalveolar lavage (BAL) cytospin CD11b and Gr1 immunostaining. Manually thresholded BAL cytospins from Groups I‐III were analyzed for (a) Perimeter (µm) to assess the size of BAL cells. (b) Percent CD11b positive, negative, Gr1 positive, negative and double‐negative cells. (c) Maximum (Max) CD11b fluorescent intensity per CD11b positive cell. (d) CD11b fluorescent integrated density (Normalized CD11b expression) (e) CD11b skewness to assess the frequency distribution of high versus low pixel intensities on each positive cell. (f) CD11b kurtosis to assess the spread of pixel frequency distribution. G Standard deviation (S.D.) in the CD11b fluorescent intensity to assess the variation in pixel intensities on each cell. (h) Maximum (Max) Gr1 fluorescent intensity per Gr1 positive cell. (i) Gr1 fluorescent integrated density (Normalized Gr1 expression). (j) Gr1 skewness to assess the frequency distribution of high versus low pixel intensities on each positive cell. (k) Gr1 kurtosis to assess the spread of pixel frequency distribution. (l) Standard deviation (*SD*) in the Gr1 fluorescent intensity to assess the variation in pixel intensities on each cell. *p < .05, **p < .01 represent pair‐wise statistical significance. The data points for each labeled protein depict multiple cells analyzed in three mice. Data are analyzed with a two‐way ANOVA after averaging the multiple cells from a single mouse and analyzing the fluorescent read‐outs from three mice

0.05 ppm O_3_ (Group III) exposure for 2 hr induced recruitment of larger BAL cells when compared to control (Group I) or 0.005 ppm of O_3_ exposure for 2 hr (Group II) (Figure 5a). More than 95% of the BAL cells were CD11b‐ and Gr1‐positive cells in the control Group I (Figure 5b). Both 0.005 and 0.05 ppm O_3_ exposures for 2 hr, induced a significant decrease in the total number as well as proportion of BAL CD11b (up to 40.38%, *p* < .01) and Gr1 (up to 21.27%, *p* < .01)‐positive cells (Figure 5b). It must be noted that > 95% of the Gr1‐negative population in Groups II and III were also negative for CD11b.

Among the CD11b‐positive cells, O_3_ induced recruitment of distinct (brighter) mononuclear BAL CD11b cells, especially in group III compared to control (group I, Figure S1a–c, https://figshare.com/s/83fe2fdabbc32e0d954a). The brighter CD11b cells were also larger (Figure 5a, Figure S1g, https://figshare.com/s/83fe2fdabbc32e0d954a, H https://figshare.com/s/83fe2fdabbc32e0d954a). These CD11b‐positive cells had higher maximum (Figure 5c) and total fluorescence intensity (Figure 5d), were positively skewed (i.e., a higher bright CD11b frequency distribution, as shown in Figure 5e), had higher kurtosis (i.e., a discrete CD11b spatial distribution, as shown in Group III Figure 5f), and more variation in the CD11b distribution compared to control (Figure 5g).

O_3_ exposure did not induce recruitment of more Gr1 cells when compared to control (group I) (Figure S1d,e,f, https://figshare.com/s/83fe2fdabbc32e0d954a). However, the Gr1‐positive cells had lower maxima (Figure 5h) but no change in total fluorescence intensity (Figure 5i), were positively skewed (Figure 5j), had higher kurtosis at 0.005 ppm, but not at 0.05 ppm O_3_ (Figure 5k). Interestingly, O_3_ exposed BAL cells displayed lower variation in Gr1 distribution (Figure 5l) compared to control (Figure S1g,h, https://figshare.com/s/83fe2fdabbc32e0d954a).

Thus, O_3_ did not increase the number and proportion of Gr1‐positive cells, unlike the presence of CD11b bright cells in BAL, but these cells had lower fluorescence intensity deviation and a higher frequency of bright Gr1 distribution compared to the control group I.

### 3.5 O_3_ upregulates lung CD11b, ATPβ, plasminogen, angiostatin, CD62P, and vwf expression

**FIGURE 6:**
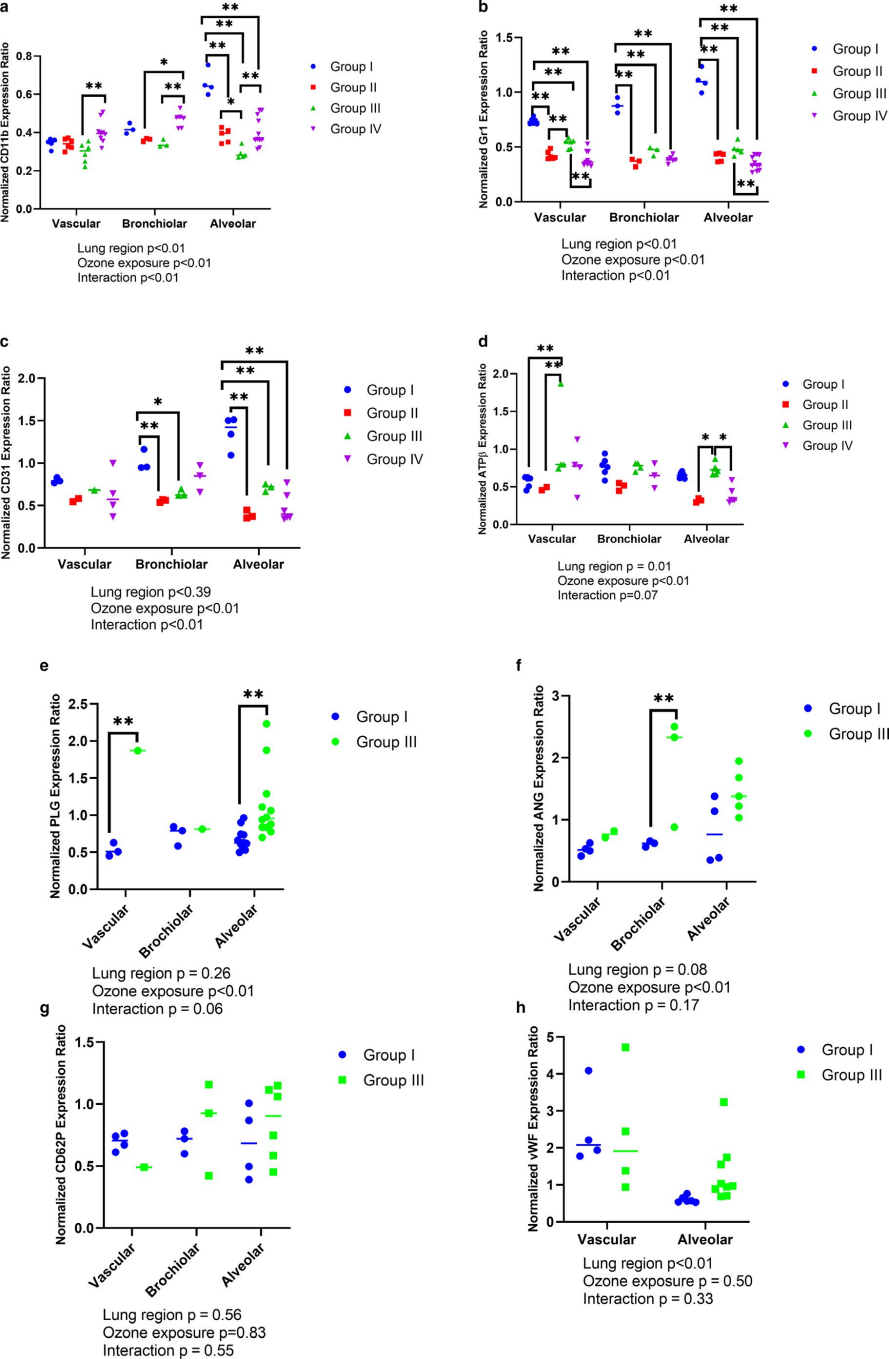
Lung immunostaining. Lung cryosections (5 µm) were analyzed for normalized expression ratio of protein markers in vascular, bronchiolar and alveolar regions for groups I‐IV for (a) CD11b, (b) Gr1, (c) CD31 (Platelet endothelial cell adhesion molecule‐1, PECAM‐1), (d) ATP synthase β subunit (ATPβ), and groups I and III for (e) Plasminogen (PLG), (f) Angiostatin (ANG), (g) P‐selectin (CD62P), and (h) von Willebrand factor (vWF). *p < .05, **p < .01 represent pair‐wise statistical significance. The data points for each labelled protein depict multiple lung regions analyzed in three mice. Data are analyzed with a two‐way ANOVA after averaging the read‐outs of multiple regions from a single mouse and analyzing the average fluorescent intensity from 3 mice

Shorter O_3_ exposures of 2 hr reduced CD11b expression in vascular, bronchiolar as well as alveolar regions (Figure 6a), with significant suppression at 0.05 ppm (Group III) (note Groups II and III had majority of CD11b positive cells in BAL). The longer (4 hr) and higher (0.5 ppm, Group IV) O_3_ exposure led to significant CD11b expression in the vascular and bronchiolar, but not the alveolar regions, when compared to control (Group I) (Figure 6a, Figure S2a https://figshare.com/s/83fe2fdabbc32e0d954a). The alveolar CD11b expression was the least after short 0.05 ppm (Group III) O_3_ exposures when compared to baseline (Group I), or after short 0.005 ppm (Group II) (Figure 6a).The alveolar CD11b expression was higher after 4 hr of 0.5 ppm exposure (Group IV) compared to Group II (Figure 6a). As longer O_3_ exposures yielded negligible BAL cell recovery, this is also, why we did not perform IF BAL CD11b and Gr1 staining in groups IV and V. The lung CD11b quantification shows that there is a reciprocal relationship between BAL and lung CD11b‐positive cells. The percentage of CD11b‐positive cells that are recovered from BAL are correspondingly absent from the alveolar region, after 2 hr of 0.005 or 0.05 ppm O_3_ exposure.

O_3_ at 0.005 (Group II) or 0.05 ppm (Group III) for 2 hr and 0.5 ppm for 4 hr (Group IV) significantly blocked Gr1 expression in all the lung regions (Figure 6b, Figure S2a https://figshare.com/s/83fe2fdabbc32e0d954a) when compared to control (Group I).

CD31 is an adhesion molecule expressed on endothelial cells as well as some leukocytes. The vascular CD31 expression was not significantly affected by O_3_ exposures when compared to the control group I (Figure 6c). Alveolar and bronchiolar CD31 expression were reduced after O_3_ exposures, when compared to control group I. However, in the bronchiolar region, longer, that is, 4 hr 0.5 ppm O_3_ exposure, did not affect CD31 protein expression (*p* > .05, Figure 6c, Figure S2b, https://figshare.com/s/83fe2fdabbc32e0d954a).

O_3,_ exposure at 0.05 ppm for 2 hr (Group III), enhanced vascular ATPβ (Figure 6d, Figure S2b, https://figshare.com/s/83fe2fdabbc32e0d954a), vascular and alveolar plasminogen (Figure 6e, Figure S2c, https://figshare.com/s/83fe2fdabbc32e0d954a) and bronchiolar angiostatin (Figure 6f, Figure S2d,https://figshare.com/s/83fe2fdabbc32e0d954a) normalized fluorescence. ATPβ is predominantly localized in endothelial cells, and is elevated by fourfold after 0.05 ppm of O_3_ exposure for 2 hr (Figure 4d, *p* < .01). An interesting pattern was observed in the alveolar region where 2 hr exposure to 0.05 ppm O_3_ enhanced the alveolar ATPβ intensity while 4 hr exposure to 0.005 ppm O_3_ reduced the alveolar ATPβ fluorescence intensity (Figure 4d), likely due to extensive alveolar damage. Group III CD62P (Figure 6g, Figure S2e, https://figshare.com/s/83fe2fdabbc32e0d954a) and vwf (Figure 6h, Figure S2f, https://figshare.com/s/83fe2fdabbc32e0d954a) fluorescent intensities, showed large standard deviation, when compared to control Group I (in vascular as well as alveolar regions).

Thus, lung CD11b and Gr1 protein quantification corroborates our BAL findings. Interestingly, we observed an increase in the bronchiolar expression of angiostatin, while plasminogen and ATP synthase β subunits were observed largely in the vascular and alveolar regions.

### 3.6 O_3_ induces an IL‐16 predominant inflammatory response in mice

**FIGURE 7:**
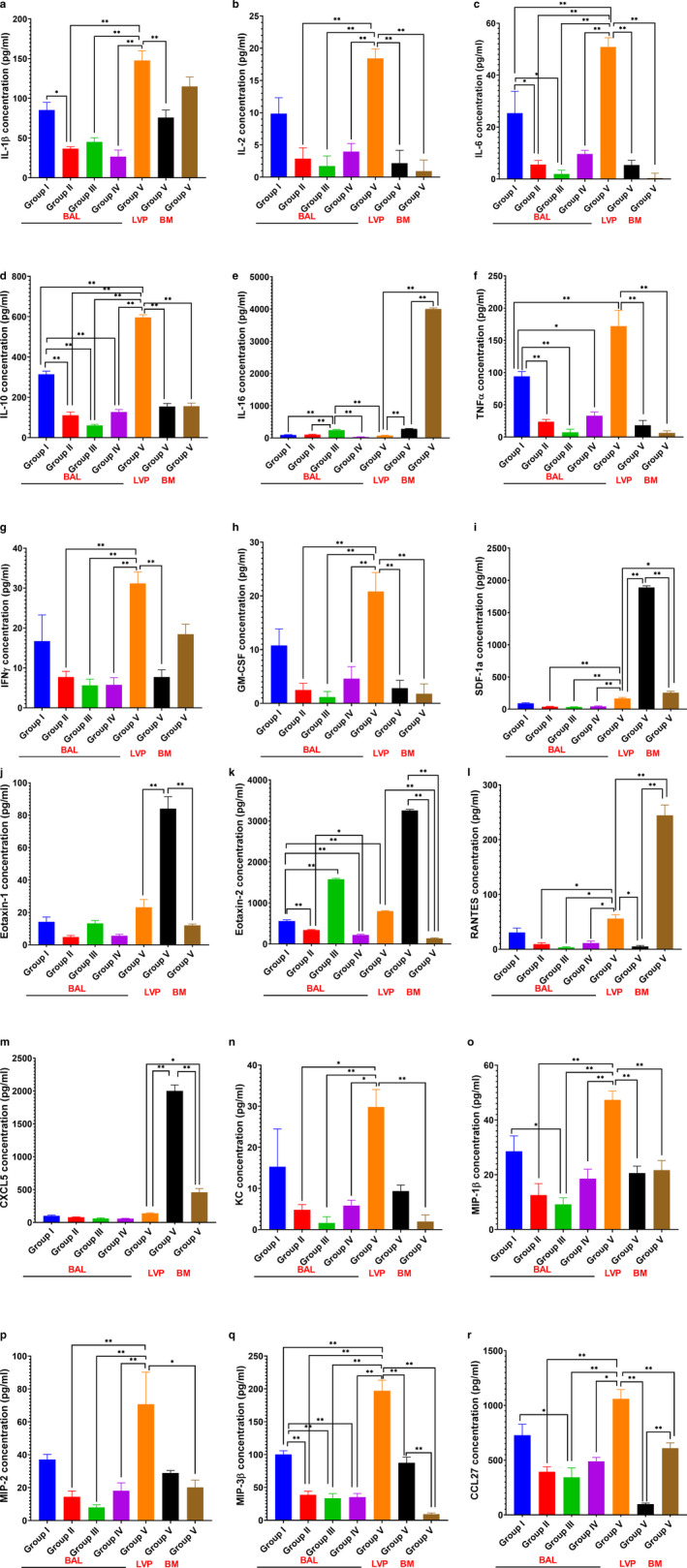
Ozone (O_3_)‐induced lung (bronchoalveolar lavage (BAL), lung vascular perfusate, and bone marrow) chemokine 33‐plex analysis. A Concentrations (pg/ml) of the 18 of 33 chemokines analyzed in bronchoalveolar lavage (BAL) fluid from groups I‐V, lung vascular perfusate (LVP) and bone marrow (BM) from group V: (a) IL‐1β, (b) IL‐2, (c) IL‐6, (d) IL‐10, (e) IL‐16, (f) TNFα, (g) IFNγ, (h) GM‐CSF, (i) SDF‐1a, (j) Eotaxin‐1, (k) Eotaxin‐2, (l) RANTES, (m) CXCL5, (n) KC, (o) MIP‐1β, (p) MIP‐2, (q) MIP‐3β, and (r) CCL27. BAL from group I is shown in blue, group II BAL in red, group III BAL in green, group IV BAL in purple, group V BAL in orange, group V LVP in black and group V BM in brown bar graphs. Data are analyzed by one‐way ANOVA and the *p*‐value for false discovery rate of multiple variables was adjusted as per Benjamini and Hoshberg correction. Pair‐wise comparisons were analyzed after Bonferroni's correction. * represents *p* < .05, ** represents *p* < .01

Short O_3_ exposures (0.005, 0.05 ppm for 2 hr) led to the release of an eosinophil chemotaxin, eotaxin‐2 (4.67 fold) (Figure 7k, Figure S3, https://figshare.com/s/83fe2fdabbc32e0d954a) and interleukin, IL‐16 (2.66 fold, Figure 7e) in BAL when compared to group I. We also observed deformed eosinophils in O_3_ exposed BAL cytospins from groups II‐V. However, the numbers did not reach significance compared to control (Group I).

2 hr of 0.05 ppm O_3_ exposure reduced IL‐6 (Figure 7c), IL‐10 (Figure 7d), TNFα (Figure 7f), RANTES (Figure 7l), KC (Figure 7n), CCL27 (Figure 7r), MIP‐1β (Figure 7o), and MCP1 (Figure S4i, https://figshare.com/s/83fe2fdabbc32e0d954a) concentrations in BAL when compared to group I. An increase in exposure time from 2 (group II) to 24 hr (group V), at 0.005 ppm O_3,_ released both immuno‐suppressive as well as stimulant chemokines in BAL, like IL‐1β (3.28 fold, Figure 7a), IL‐2 (10.58 fold, Figure 7b), IL‐6 (26.08 fold, Figure 7c), IL‐10 (9.81 fold, Figure 7d), TNFα (23.49 fold, Figure 7f), IFNγ (5.53 fold, Figure 7g), GM‐CSF (17.49 fold, Figure 7h), SDF‐1a (4.45 fold, Figure 7i), RANTES (5.95 fold, Figure 7l), CXCL5 (Figure 7m), KC (18.18 fold, Figure 7n), MIP‐1β (3.77 fold, Figure 7o), MIP2 (4.91 fold, Figure 7p),MIP3β (5.08 fold, Figure 7q), CCL‐27 (2.69 fold, Figure 7r), eotaxin‐1 (Figure 7j), CXCL10 (2.45 fold, Figure S4a, https://figshare.com/s/83fe2fdabbc32e0d954a), CXCL11 (8.07 fold, Figure S4b), MCP‐1 (14.79 fold, Figure S4i, https://figshare.com/s/83fe2fdabbc32e0d954a), CX3CL1 (6.84 fold, Figure S4f, https://figshare.com/s/83fe2fdabbc32e0d954a), TARC (3.19 fold, Figure S4g, https://figshare.com/s/83fe2fdabbc32e0d954a), TECK (2.87 fold, Figure. S4h, https://figshare.com/s/83fe2fdabbc32e0d954a) and (Figure S3, https://figshare.com/s/83fe2fdabbc32e0d954a, Figure 7a).

To assess the chemokine gradients established in group V, we also assessed the chemokines in lung vascular perfusate and the bone marrow. Concentrations of CXCL5 (3.34 fold, Figure 7m), IL‐16 (53.68 fold, Figure 7e), RANTES (4.38 fold, Figure 7l), SDF‐1a (1.55 fold, Figure 7i) were highest in the bone marrow compartment, when compared to the BAL and vascular compartments (Figure S3, https://figshare.com/s/83fe2fdabbc32e0d954a). Remarkably, the concentrations of pan leukocyte chemokine, SDF‐1a (Figure 7i), mononuclear cell chemokines, CXCL10 (Figure S4a, https://figshare.com/s/83fe2fdabbc32e0d954a), CXCL13 (Figure S4c, https://figshare.com/s/83fe2fdabbc32e0d954a), the neutrophil chemokine CXCL5 (Figure 7m) and the eosinophil chemokines eotaxin‐1 (Figure 7j) and eotaxin‐2 (Figure 7k), were highest in lung vascular perfusate after 0.005 ppm O_3_ exposures for 24 hr.

Thus, even 0.005 ppm of O_3_ exposure, for 24 hr, induce the release of BAL IL‐1 dependent, chemokines, bone marrow IL‐16 and RANTES and lung vascular SDF‐1a, eotaxin‐1, −2, and CXCL5. Short 2 hr exposures to 0.05 ppm O_3_ induced release of BAL IL‐16 and eotaxin‐2, thus highlighting neutrophil, eosinophil, and platelet cell dysregulation.

### 3.7 0.05 ppm O_3_ induces a heterogeneous loss of sub‐pleural alveolar cytockeletal actin and dysregulated ventilation

**FIGURE 8:**
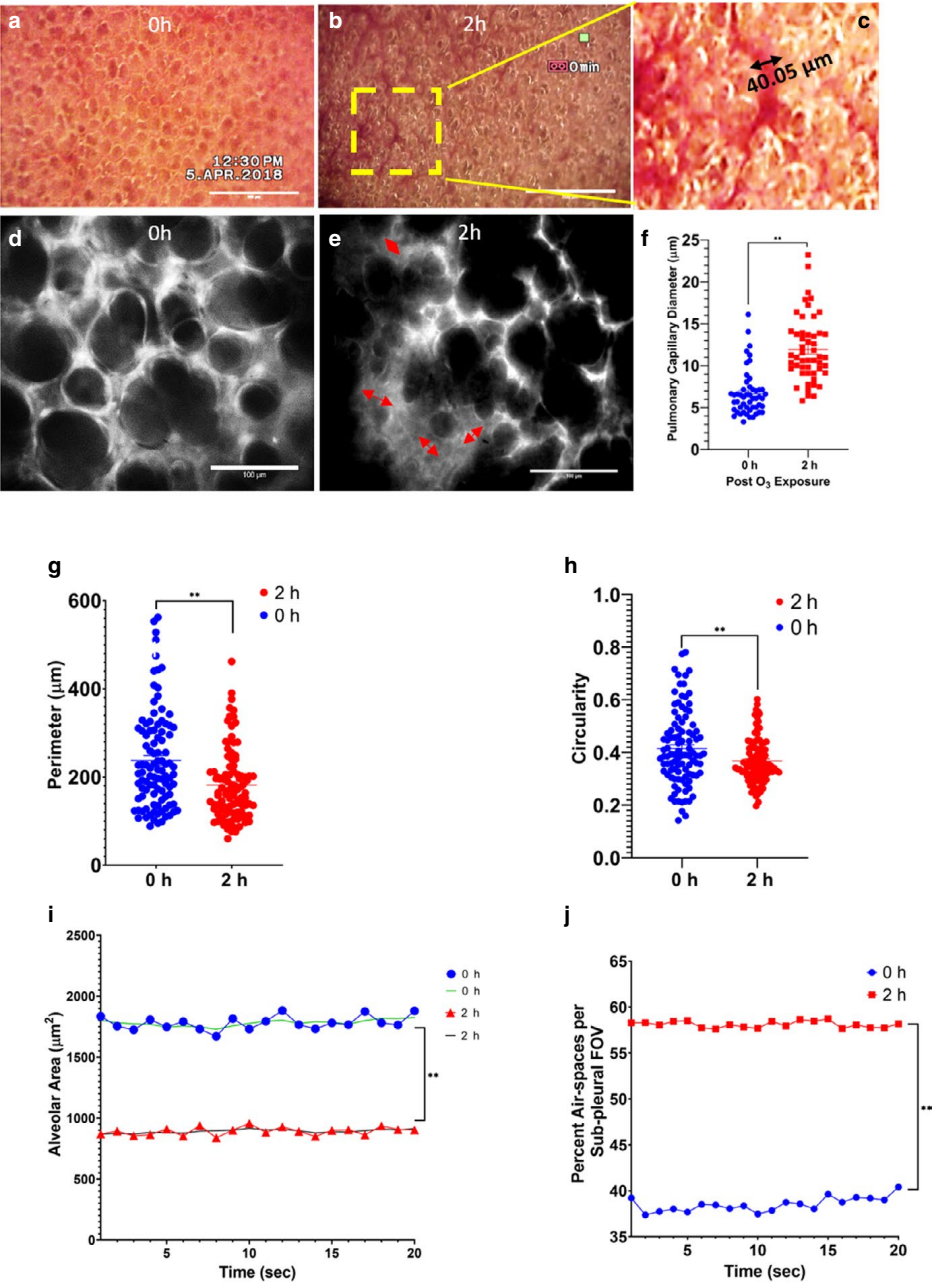
Ozone (O_3_)‐induced alterations in in situ alveolar actin morphology. Representative stabilized lung thoracic window RGB images of sub‐pleural C57BL/6NJ mice bright‐field (without any dye or antibody treatment) exposed to (a) room air (i.e., 0 hr or control) or (b) 2 hr 0.05 ppm O_3_. (c) Note the magnified view of the rectangular field from (b) showing marked vasodilatation (40.05 µm capillary diameter) in response to 0.05 ppm O_3_. Representative 40× views of rhodamine phalloidin stained red fluorescent emission filtered light alveolar images visualized through ICCD camera (Stanford Photonics XR/MEGA 10 Extreme) in C57BL/6NJ mice exposed to (d) 0 hr (i.e., room air), showing well‐organized septal alveolar actin filaments or (e) 2 hr 0.05 ppm O_3_, showing heterogeneously damaged alveolar regions stretched out as a membrane. The diffuse contractile actin is shown by red double edged arrows which denote the diffuse actin morphology and numerous pores of Kohn or open circular air spaces are marked by yellow asterisks (*). (f) Distribution of pulmonary capillary diameter (μm) before (0 hr, shown as blue scatter plots) and after (shown as red scatter plots) 0.05 ppm ozone (2 hr) exposure, ** represents *p* < .01. (g) Distribution of alveolar perimeter (μm) before (0 hr, shown as blue scatter plots) and after (shown as red scatter plots) 0.05 ppm ozone (2 hr) exposure, (h) Alveolar space circularity before (0 hr, shown as blue scatter plots) and after (shown as red scatter plots) 0.05 ppm ozone (2 hr) exposure *p < .05, ** *p* < .01. *N* = 3 per group. (i) Representative line plots showing changes in alveolar area (μm^2^) of a single alveolus over 20 respiratory cycles, before (0 hr, shown in blue for original data points and green for smoothened data points) and after (shown in red for original data points and purple for smoothened data points) 0.05 ppm ozone (2 hr) exposure. ** represents *p* < .01. (j) Representative line plots showing percentage of area occupied by air‐spaces in one sub‐pleural field of view (FOV) over 20 respiratory cycles, before (0 hr, shown in blue) and after (shown in red) 0.05 ppm ozone (2 hr) exposure. ** represents *p* < .01

Intravital lung microscopy from experiment 2 revealed pulmonary capillary vasodilatation immediately after 0.05 ppm O_3_ exposure (median diameter of 10.99 μm, Figure 8b,c,f) compared to the control group (median diameter of 6.10 µm, *p* < .01, Figure 8a). We superfused phalloidin to observe the alveolar actin arrangement at 0, 2, and 24 hr after 0.05 ppm O_3_ exposure. At 0 hr, we observed peripherally organized contractile actin filaments around alveolar spaces (Figure 8d and Figure S5a, https://figshare.com/s/83fe2fdabbc32e0d954a). Moreover we observed the collective rhythmic motion of these filaments during respiration (Video 1 https://figshare.com/s/0b26c16f50f8dafef4c1, Figure 8h). O_3_ exposure led to heterogeneous alveolar damage (Figure 8e, Figure S5b‐d, https://figshare.com/s/83fe2fdabbc32e0d954a) marked by an increase in alveolar or air‐space perimeter (Figure 8g) and reduction in circularity (Figure 8h). The sub‐pleural alveoli showed either diffuse, discontinuous actin with lots of pores (Figure 8j, Figure S5b‐d, https://figshare.com/s/83fe2fdabbc32e0d954a), or extremely thin and condensed stress fibers (Figure 8e, Videos 2 https://figshare.com/s/0b26c16f50f8dafef4c1 and 3 https://figshare.com/s/0b26c16f50f8dafef4c1). Video‐microscopy revealed that O_3_ prolongs inspiratory alveolar distention (Videos 2 https://figshare.com/s/0b26c16f50f8dafef4c1 and 3 https://figshare.com/s/0b26c16f50f8dafef4c1, Figure 8h). Alveolar area analysis, shown for 20 respiratory cycles, revealed a twofold reduction in net displacement of a representative alveolar area as shown in Figure 8i. O_3_ also caused a twofold reduction in the average fluorescence intensity of actin (phalloidin) (Figure S5e, https://figshare.com/s/83fe2fdabbc32e0d954a).

### 3.8 0.05 ppm O_3_ induces immediate loss of sub‐pleural ROS and mitochondrial potential followed by resurgence at 24 hr

**FIGURE 9:**
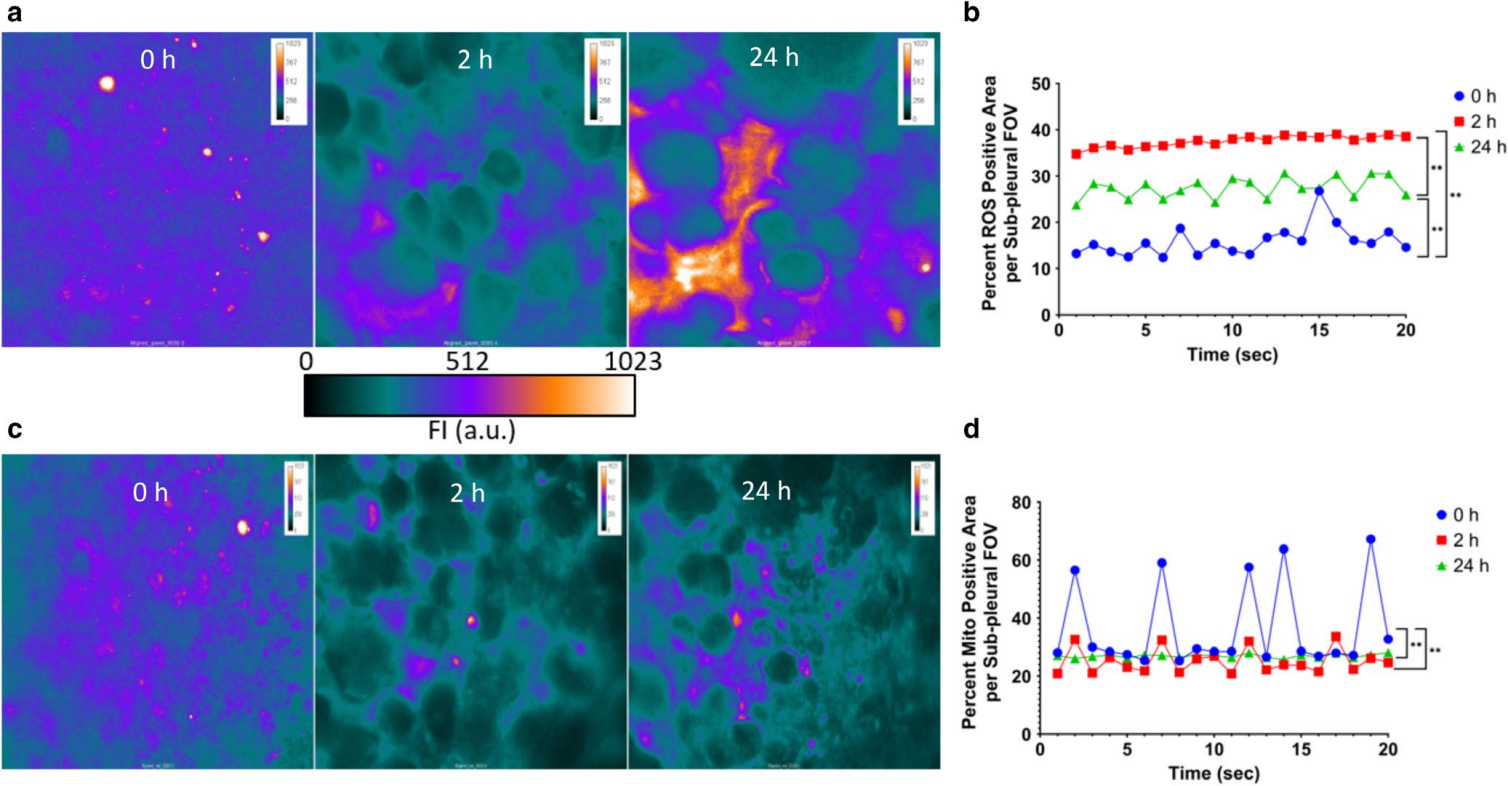
Ozone (O_3_)‐induced lung intravital reactive oxygen species (ROS) and mitochondrial potential (reduced mitotracker) imaging. (a) Representative 40× color scaled images of ROS, at 0, 2, and 24 hr post 0.05 ppm ozone exposure. (b) Representative plot profiles of percentage of alveolar area positive for ROS activity in one sub‐pleural field of view (FOV), for 20 respiratory cycles. Plot profiles are shown as blue for 0 hr, red for 2 hr and green for 24 hr. *N* = 3 per group. ** represents *p* < .01. (c) Representative 40× color scaled images of reduced mitotracker, at 0, 2, and 24 hr post 0.05 ppm ozone exposure. (d) Representative plot profiles of percentage of the alveolar area positive for reduced mitotracker activity in one sub‐pleural field of view (FOV), for 20 respiratory cycles. Plot profiles are shown as blue for 0 hr, red for 2 hr, and green for 24 hr. *N* = 3 per group. ** represents *p* < .01

In situ labeling of lungs for ROS, which is a marker of total cellular ROS generation and oxidative stress, showed several important features. Under baseline or RA exposure, specific lung parenchymal cells stain positive for ROS as indicated by significantly high activity in circular cells (Figure 9a). However, it is worth noting that these ROS‐positive cells comprise 10%–20% of the parenchymal area (Figure 9b). Although there is not a significant difference in ROS‐positive area before or after O_3_ exposure, the activity is representative of the respiratory cycles at 0 and 24h. ROS activity was also higher at 0 hr when measured as difference in ROS (green channel) signal over time (10 min, T1‐T2) (Figure S6a, https://figshare.com/s/83fe2fdabbc32e0d954a, b,c) and maximum intensity in the green channel (Figure 9a, Figure S7d) compared to at 2 hr O_3_ exposure. 35% of the lung parenchyma was ROS positive (Figure 9b). At 24 hr after 2 hr 0.05 ppm O_3_ exposure, lungs regain their ability to generate ROS (Figure 9a and Figure S6a‐c, https://figshare.com/s/83fe2fdabbc32e0d954a).

A similar pattern was observed with in situ reduced mitotracker lung staining (red channel) of lung alveolar cells. However, 20%–60% of the parenchymal cells are positive for reduced mitotracker dye at baseline (Figure 9c,d Video 4 https://figshare.com/s/0b26c16f50f8dafef4c1) and show a complete block of activity (Figure 9c, Figure S7a, https://figshare.com/s/83fe2fdabbc32e0d954a) and maximum mitotracker dye staining (Figure 9c, Video 5 https://figshare.com/s/0b26c16f50f8dafef4c1, Figure S7a–d, https://figshare.com/s/83fe2fdabbc32e0d954a) after 2 hr O_3_ exposure to 0.05 ppm O_3_. At 24 hr after 2 hr 0.05 ppm O_3_ exposure, 20%–30% of the parenchymal cells were distinctly positive for reduced mitotracker; this points to regeneration of redox potential (Figure 9c,d, Figure S7a–d Video 6 https://figshare.com/s/0b26c16f50f8dafef4c1). We confirmed our live actin, ROS and mitotracker findings by staining cytospins of the BAL (Figure S8a‐c, https://figshare.com/s/83fe2fdabbc32e0d954a), lung vascular perfusate as well as peripheral blood cells (data not shown).

Our intravital observations show loss of inter‐septal connective tissue, compromised respiratory motion, immediately after 0.05 ppm of 2 hr O_3_ exposure. This is also accompanied by a block of baseline cellular levels of ROS and mitochondrial potential. However, room air exposure of up to 22 hr after O_3_ exposure regains the levels of ROS and mitochondrial potential in the alveolar sepal region.

## 4 DISCUSSION

**FIGURE 10:**
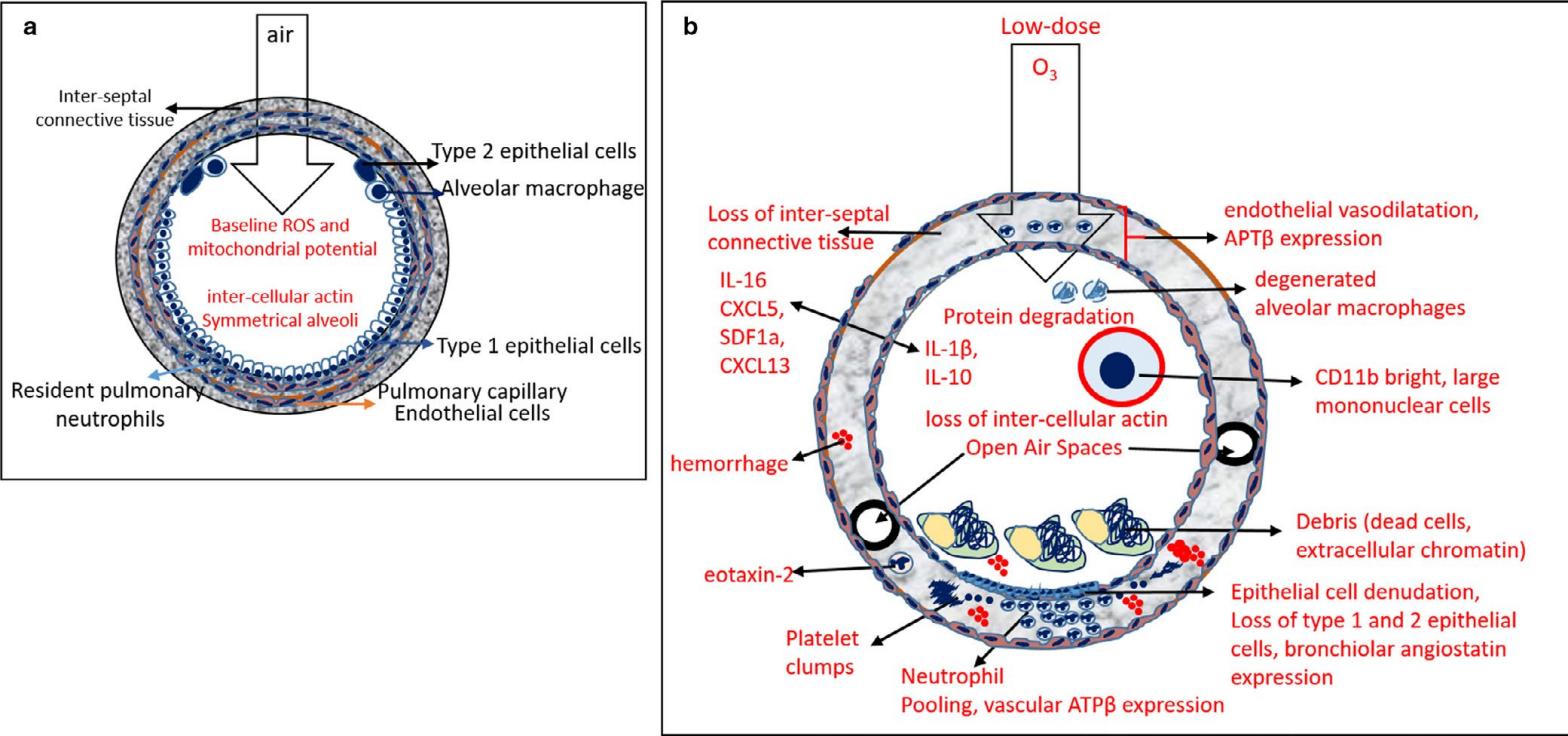
Schematic of the lung response to low‐dose ozone (a) Normal alveolar structure exposed to air showing thin alveolar walls comprised of healthy bronchiolar epithelium (type 1 and 2 cells), alveolar macrophages, inter‐alveolar actin, pulmonary capillary, and resident neutrophils, all of which have active mitochondria and ROS production at baseline levels. (b) Ozone (O_3_) exposed alveolus showing response to low doses (0.005, 0.05, 0.5 ppm). The bronchiolar epithelium is denuded (positive for angiostatin), alveolar macrophages are degenerated, the pulmonary capillaries are dilated, neutrophils get pooled in capillaries and are juxtaposed to the denuded epithelium. Platelets, debris and extracellular chromatin are prevalent in the vasculature as well as alveolar space, which account for loss of active mitochondria, ROS. The vasculature is positive for ATP synthase β sub‐unit and plasminogen. The alveolar wall loses inter‐alveolar actin but smaller circular air‐spaces are prevalent. Majority of these dead cells release immune‐stimulatory (IL‐1β, TNFα, IL‐6, IL‐16) as well as suppressive (IL‐10) chemokines and stimulate the release of chemokines such as CXCL5 (neutrophils), eotaxin‐2 (eosinophils), RANTES (both neutrophils and eosinophils), and SDF1a (pan leukocyte). Along with the vascular and alveolar chemokines, large CD11b positive mononuclear cells are observed instead, in the alveolar space, which might account for rejuvenation of the active mitochondria and ROS levels

Our current study shows that low‐dose O_3_ exposure (i.e., below 0.063 ppm) is capable of inducing acute lung injury and systemic inflammation in C57BL/6NJ mice. Our model presents sensitive read‐outs to assess sterile lung inflammation as summarized in a schematic format in Figure 10.

Systematic analysis of the BAL, lung vascular perfusate, and peripheral blood compartments revealed compartment‐specific effects of 24 hr O_3_ exposure. The short exposures led to decrease in BAL mononuclear cells, majority of which were degenerated and an increase in neutrophils in lung vascular perfusate and peripheral blood. Thus, neutrophils are recruited into the lungs but they die in the alveolar space (as indicated by their absence in recovered BAL cells).

BAL total protein content is an index of protein (airway surface liquid) secreted by the epithelial cells as well as lung edema as a result of vascular barrier disruption during inflammation. The reduction in BAL total protein in groups IV and V when compared to control group I is likely due to extensive epithelial cell death and thus loss of resulting protein secretion by airway surface liquid as noted in earlier studies (National Toxicology Program, 1994). The presence of debris in the BAL, and platelets in lung vascular perfusate and peripheral blood also translated into a higher total protein content in both the lung vascular perfusate and peripheral blood after prolonged sub‐lethal (0.005 ppm) 24 hr O_3_ exposure. Because platelets are a high source of proteins in blood (Crosley et al., 2009), our results indicate progressively increasing vascular damage and platelet dysregulation by O_3_. 4 hr 0.5 ppm O_3_ exposure induced acute cell death (as noted by cell debris in lung vascular perfusate), which translated into attenuated total protein content in peripheral blood. As well, total proteins in BAL and peripheral blood increased after exposure for 2 hr. The variable CD62P and vwf expressions in lung also indicate O_3‐_induced endothelial cell and platelet dysregulation. Overall, we observed acute alveolar cell death, which was supported by systematic findings in the BAL, lung vascular perfusate, peripheral blood as well as lung histology. Short (2 hr) O_3_ exposures induced significant bronchiolar and endothelial cell damage, which progressed to development of frank hemorrhage and fibrin clumps at longer (4 and 24 hr) O_3_ exposures, indicating endothelial dysfunction and platelet activation.

Other than acute cell death, discrete CD11b bright and large macrophages were prevalent in the BAL but absent in the lung vascular perfusate or peripheral blood (data not shown) following 0.05 ppm O_3_ exposure for 2 hr. Moreover the fact that bronchiolar and vascular lung regions had more Cd11b positive cells suggests that these cells are highly adherent, after long (4 hr) O_3_ exposures. As monocyte chemoattractants are not appreciably increased in the BAL or vascular compartment, we suspect these bright and large CD11b‐positive mononuclear cells to be derived from the visceral pleural lining macrophages (Cailhier et al., 2006). Pleural cavity cells such as macrophages and mesothelial cells are reported to follow haptokinetic migration to reach the lung parenchyma (Mubarak et al., 2012) and lymph nodes (Lehnert, 1992). Systematic studies in the future will be crucial to identify the important cell types or molecular signaling involved in the initiation of the repair processes that involve CD11b‐positive (pleural cavity, alveolar, or monocyte‐derived) macrophages.

Alveolar partitioning of neutrophils was a common feature after short and long O_3_ exposures (Figure 10b). O_3_, being a toxic chemical, instantly kills cells creating a zone of necrosis. Rapid neutrophil swarming outside the necrosis zone is typical of sterile inflammation models in tissues (McDonald et al., 2010; Wang et al., 2017). Neutrophils do eventually migrate into the necrotic zone and into the alveolar spaces but initially accumulate in the interstitial space around the alveoli. Most notably, the lung vascular perfusate comprised > 90% neutrophils after only 2 hr of 0.005 or 0.05 ppm O_3_ exposure. The fact that O_3_ did not increase the number and proportion of Gr1 positive cells in BAL, but these cells had lower fluorescence intensity deviation and a higher frequency of bright Gr1 distribution compared to the control group I, points toward phagocytosis of the dead Gr1 positive cells (neutrophils, eosinophils, monocytes, iNKT cells and macrophages). High BAL IL‐16 levels after 2 as well as 24 hr O_3_ exposures provide further evidence of secondary neutrophil necrosis. Activated macrophages, neutrophils as well as platelets release enzymes such as MMPs (matrix metalloproteinases) which degrade tissue extracellular matrix in the necrotic zone, cleave plasminogen to angiostatin fragments and clear paths for new vessels and collagen deposition (Benelli, Morini, Brigati, Noonan, & Albini, 2003; Benelli et al., 2002). In our O_3_ model, the high expression pattern of angiostatin near the bronchiolar regions, was juxtaposed to its precursor's (plasminogen) or its binding partner's (ATPβ) vascular and alveolar expression (Aulakh, 2018; Aulakh, Balachandran, Liu, & Singh, 2013). This shows that plasminogen is preferentially cleaved to angiostatin at sites of damage, like the bronchi.

Analysis of the 33‐plex chemokine panel run on BAL fluid, lung vascular perfusate and bone marrow supernatants also revealed that O_3_ induces release of both immune stimulatory (IL‐1β, IL‐2, TNFα, IL‐6, IFNγ) as well as suppressive (IL‐10) chemokines. High alveolar concentrations of CXCL10, CX3CL1, and IL‐16 (Center, Kornfeld, & Cruikshank, 1997; Ichikawa et al., 2013; Roth et al., 2015; Roth, Solbach, & Laskay, 2016), correlates with the secondary necrosis and phlogistic debris observed in the BAL and lung vascular perfusate cytospins from O_3_ exposed groups. The release of TARC and CXCL16 points toward probable activation of iNKT cells, which are capable of sensing damaged membrane phospholipids as well as releasing neutrophil chemoattractants like KC (Thanabalasuriar, Neupane, Wang, Krummel, & Kubes, 2016). A predominant release of the chemokines neutrophil as well as eosinophil chemokines such as, CXCL10, IL‐16, CXCL5, RANTES, and SDF‐1a (Garcia‐Cuesta et al., 2019; Ichikawa et al., 2013; Kawaguchi, Zhang, & Nakanishi, 2019; Pan, Parkyn, Ray, & Ray, 2000) from bone marrow points to robust inflammatory response to 0.005 ppm O_3_ concentrations. Notably, the eosinophil chemotactic agent, eotaxin‐2, is released in BAL after 2 hr 0.05 ppm O_3_ exposure, and even higher levels are observed in vascular perfusate after 24 hr 0.005 ppm O_3_ exposure, indicating the recruitment of eosinophils. We also observed eosinophils in the H&E stained cryosections of spleen and thymus from O_3_ exposed mice (data not shown).

Imaging the dynamics of lung damage is required to understand the orchestration of the lung inflammatory response to O_3_. Inflammation is a sum of the vascular response and remodeling of the surrounding tissues as observed by altered diffuse alveolar actin morphology showing numerous pores in the alveoli, which are probably the pores of Kohn (Scarpelli, 2003). In addition, the lung video sequences showed perturbed respiratory motion as a direct evidence of O_3_‐induced mechanical lung damage. Similar actin cytoskeletal changes have been reported secondary to cigarette smoke‐induced damage to the alveolar epithelial cell monolayer (Nishida et al., 2017). Our observations of lung ROS and reduced mitotracker in live animals are also in agreement with the ex vivo ROS, mitotracker and actin staining of BAL, perfusate and peripheral blood cells. Whereas immediately after O_3_ exposure, the pulmonary epithelium is not mitochondrially active and incapable of producing ROS, O_3_ damaged tissue adapts by regaining mitochondrial sensitivity and ROS as seen in mice exposed to 0.05 ppm O_3_ for 2 hr and recovered for 22 hr under room air. Although live imaging showed that immediately after O_3_ exposure, when lung parenchymal cells are mitochondrially inactive and do not produce ROS, the lung tissue showed enhanced immune‐fluorescent vascular lung ATP synthase β subunit (ATP β) expression and neutrophils concentrated within the lung vasculature, and not alveolar air sacs (as indicated by cell counts). At 24 hr after O_3_ exposure, when the lung tissue regains its ROS and mitochondrial potential, the lung vascular neutrophils plateau out and alveolar macrophage recruitment takes over.

There are numerous reports on the effects of much higher (1–2 ppm) concentrations of O_3_ exposure at 24 hr but these studies might be overexposing the mice and not capturing the primal inflammatory events, after O_3_ exposures (Francis et al., 2017; Tighe et al., 1950). Our results present a controlled model to study the acute cellular and tissue response to increasing concentration and time of O_3_ exposures. Radiation induced lung injury is also characterized by reduction in ROS, mitochondrial shrinkage due to excessive lipid peroxidation and ferroptotic cell death (Li, Zhuang, & Qiao, 2019). Our experiments have captured an important, but often ignored, role of ROS and mitochondrial potential in normal cellular signaling and tissue hemodynamics. One limitation of our study is that we did not examine sex‐specific effects of O_3_. Although not ideal, the male sex was chosen to reduce variation in experimental data due to fewer animals per group. Therefore, future work needs to examine if the female mice are also as sensitive to deleterious effects of O_3_ as males were in our work.

The current experiments were performed on the NJ mouse sub‐strain because many commercially available knock‐out mice are on a mixed background with varying genetic contributions from both C57BL/6J and C57BL/6N sub‐strains. We were unable to image the lung vasculature, immediately after O_3_ exposure, as the NJ sub‐strain mice used in our current study, possessed frail hemodynamics. Currently, we are imaging the J sub‐strain mice.

O_3_ injury of lung epithelial cells, and the ensuing inflammatory response, which leads to the production of numerous cytokines and chemokines, recruitment of neutrophils and activation of platelets likely contributes to the capacity of O_3_ to trigger asthma, chronic obstructive pulmonary disease (COPD) (Broeckaert et al., 1999; Broeckaert, Clippe, Wattiez, Falmagne, & Bernard, 2003). It is a common component of urban smog and levels of O_3_ in smog can easily reach up to 0.05 ppm; hence the reason why we chose this concentration range in our experiments. Coping with these effects on a daily basis can pose significant burden to additional particulate and infectious exposures like LPS, bacteria, and viruses which can lead to activation of the baseline immune response. Further understanding of how repair mechanisms take effect on a chronic basis can lead to better public health policies and therapeutic management of O_3_‐induced adverse health effects.

In summary, this research describes a sensitive model of sterile murine lung inflammation induced by exposing mice to short and low levels of O_3_ that are below the current recommendations for safe ambient ozone concentration for humans. O_3_ led to concentration and time‐dependent phlogistic cell death in the broncho‐alveolar lavage, lung epithelial damage and hemorrhage. We recorded CD11b positive cells in the broncho‐alveolar lavage, upregulation of plasminogen and ATP synthase expression in the lung vascular and alveolar compartments while angiostatin was observed in the bronchiolar region in mice after 2 hr of O_3_ exposure at 0.05 ppb. We also observed platelet and neutrophil accumulation in the lung vasculature and an eotaxin‐2, IL‐16, CXCL5, CXCL12, and CXCL13 dominant inflammatory response leading to lung injury. O_3_ induced actin filament disorganization, perturbed respiratory mechanics, acute suppression of the alveolar reactive oxygen species (ROS) production and mitochondrial potential in ventilated lungs. We present evidence of systemic, as well as pulmonary toxicity, at 40‐fold lower O_3_ concentrations than previously reported in mice. Our findings may have important health implications of ozone exposure even at levels previously thought safe for humans.

## CONFLICT OF INTEREST

We have no conflict of interest to declare.

## AUTHOR CONTRIBUTIONS

GKA, ES, and JS conceived the study, designed the experiments and wrote the manuscript. GKA, JABD, and CMGS executed the experiments. GKA and JS analyzed the data. All authors have edited and approved the manuscript.

## References

[phy214463-bib-0001] Aulakh, G. K. (2018). Neutrophils in the lung: "the first responders". Cell and Tissue Research, 371, 577–588. 29250746 10.1007/s00441-017-2748-z

[phy214463-bib-0002] Aulakh, G. K. , Balachandran, Y. , Liu, L. , & Singh, B. (2013). Angiostatin inhibits activation and migration of neutrophils. Cell and Tissue Research. 10.1007/s00441-013-1753-0 24297047

[phy214463-bib-0003] Bhattacharya, J. , & Westphalen, K. (2016). Macrophage‐epithelial interactions in pulmonary alveoli. Semin Immunopathol., 38(4), 461–469. 10.1007/s00281-016-0569-x 27170185 PMC5018989

[phy214463-bib-0004] Benelli, R. , Morini, M. , Brigati, C. , Noonan, D. M. , & Albini, A. (2003). Angiostatin inhibits extracellular HIV‐Tat‐induced inflammatory angiogenesis. International Journal of Oncology, 22, 87–91. 10.3892/ijo.22.1.87 12469189

[phy214463-bib-0005] Benelli, R. , Morini, M. , Carrozzino, F. , Ferrari, N. , Minghelli, S. , Santi, L. , … Albini, A. (2002). Neutrophils as a key cellular target for angiostatin: Implications for regulation of angiogenesis and inflammation. FASEB Journal : Official Publication of the Federation of American Societies for Experimental Biology, 16, 267–269. 11772950 10.1096/fj.01-0651fje

[phy214463-bib-0006] Berman, M. E. , & Muller, W. A. (1950). Ligation of platelet/endothelial cell adhesion molecule 1 (PECAM‐1/CD31) on monocytes and neutrophils increases binding capacity of leukocyte CR3 (CD11b/CD18). Journal of Immunology, 154(299–307), 1995. 7995949

[phy214463-bib-0007] Bouthillier, L. , Vincent, R. , Goegan, P. , Adamson, I. Y. , Bjarnason, S. , Stewart, M. , … Kumarathasan, P. (1998). Acute effects of inhaled urban particles and ozone: Lung morphology, macrophage activity, and plasma endothelin‐1. The American Journal of Pathology, 153, 1873–1884. 9846977 10.1016/S0002-9440(10)65701-X PMC1866316

[phy214463-bib-0008] Broeckaert, F. , Arsalane, K. , Hermans, C. , Bergamaschi, E. , Brustolin, A. , Mutti, A. , & Bernard, A. (1999). Lung epithelial damage at low concentrations of ambient ozone. Lancet (London, England), 353, 900–901. 10.1016/S0140-6736(99)00540-1 10093991

[phy214463-bib-0009] Broeckaert, F. , Clippe, A. , Wattiez, R. , Falmagne, P. , & Bernard, A. (2003). Lung hyperpermeability, Clara‐cell secretory potein (CC16), and susceptibility to ozone of five inbred strains of mice. Inhalation Toxicology, 15, 1209–1230. 14515223 10.1080/08958370390229889

[phy214463-bib-0010] Cailhier, J. F. , Sawatzky, D. A. , Kipari, T. , Houlberg, K. , Walbaum, D. , Watson, S. , … Hughes, J. (2006). Resident pleural macrophages are key orchestrators of neutrophil recruitment in pleural inflammation. American Journal of Respiratory and Critical Care Medicine, 173, 540–547. 10.1164/rccm.200504-538OC 16357332 PMC2662938

[phy214463-bib-0011] Cakmak, S. , Hebbern, C. , Pinault, L. , Lavigne, E. , Vanos, J. , Crouse, D. L. , & Tjepkema, M. (2018). Associations between long‐term PM2.5 and ozone exposure and mortality in the Canadian Census Health and Environment Cohort (CANCHEC), by spatial synoptic classification zone. Environment International, 111, 200–211. 10.1016/j.envint.2017.11.030 29227849

[phy214463-bib-0012] Center, D. M. , Kornfeld, H. , & Cruikshank, W. W. (1997). Interleukin‐16. The International Journal of Biochemistry & Cell Biology, 29, 1231–1234. 10.1016/S1357-2725(97)00053-8 9451819

[phy214463-bib-0013] Crosley, L. K. , Duthie, S. J. , Polley, A. C. , Bouwman, F. G. , Heim, C. , Mulholland, F. , … de Roos, B. (2009). Variation in protein levels obtained from human blood cells and biofluids for platelet, peripheral blood mononuclear cell, plasma, urine and saliva proteomics. Genes & Nutrition, 4, 95–102. 10.1007/s12263-009-0121-x 19408033 PMC2690729

[phy214463-bib-0014] Dauchet, L. , Hulo, S. , Cherot‐Kornobis, N. , Matran, R. , Amouyel, P. , Edme, J. L. , & Giovannelli, J. (2018). Short‐term exposure to air pollution: Associations with lung function and inflammatory markers in non‐smoking, healthy adults. Environment International, 121, 610–619. 30312964 10.1016/j.envint.2018.09.036

[phy214463-bib-0015] Delfino, R. J. , Murphy‐Moulton, A. M. , Burnett, R. T. , Brook, J. R. , & Becklake, M. R. (1997). Effects of air pollution on emergency room visits for respiratory illnesses in Montreal, Quebec. American Journal of Respiratory and Critical Care Medicine, 155, 568–576. 10.1164/ajrccm.155.2.9032196 9032196

[phy214463-bib-0016] Dowell, A. R. , Lohrbauer, L. A. , Hurst, D. , & Lee, S. D. (1970). Rabbit alveolar macrophage damage caused by in vivo ozone inhalation. Archives of Environmental Health, 21, 121–127. 10.1080/00039896.1970.10667208 4247450

[phy214463-bib-0017] Erickson, M. A. , Jude, J. , Zhao, H. , Rhea, E. M. , Salameh, T. S. , Jester, W. , … Jordan‐Sciutto, K. L. (2017). Serum amyloid A: An ozone‐induced circulating factor with potentially important functions in the lung‐brain axis. FASEB Journal : Official Publication of the Federation of American Societies for Experimental Biology, 31, 3950–3965. 28533327 10.1096/fj.201600857RRR PMC5572691

[phy214463-bib-0018] Falcone, D. J. , Khan, K. M. F. , Layne, T. , & Fernandes, L. (1998). Macrophage formation of angiostatin during inflammation a byproduct of the activation of plasminogen. Journal of Biological Chemistry, 273, 31480–31485. 10.1074/jbc.273.47.31480 9813061

[phy214463-bib-0019] Francis, M. , Groves, A. M. , Sun, R. , Cervelli, J. A. , Choi, H. , Laskin, J. D. , & Laskin, D. L. (2017). Editor's highlight: CCR2 regulates inflammatory cell accumulation in the lung and tissue injury following ozone exposure. Toxicological Sciences : An Official Journal of the Society of Toxicology, 155, 474–484. 27837169 10.1093/toxsci/kfw226 PMC5291213

[phy214463-bib-0020] Fry, R. C. , Rager, J. E. , Zhou, H. , Zou, B. , Brickey, J. W. , Ting, J. , … Alexis, N. E. (2012). Individuals with increased inflammatory response to ozone demonstrate muted signaling of immune cell trafficking pathways. Respiratory Research, 13, 89 10.1186/1465-9921-13-89 23033980 PMC3607990

[phy214463-bib-0021] Garcia‐Cuesta, E. M. , Santiago, C. A. , Vallejo‐Diaz, J. , Juarranz, Y. , Rodriguez‐Frade, J. M. , & Mellado, M. (2019). The role of the CXCL12/CXCR4/ACKR3 axis in autoimmune diseases. Frontiers in Endocrinology, 10, 585 10.3389/fendo.2019.00585 31507535 PMC6718456

[phy214463-bib-0022] Grommes, J. , Alard, J. E. , Drechsler, M. , Wantha, S. , Morgelin, M. , Kuebler, W. M. , … Soehnlein, O. (2012). Disruption of platelet‐derived chemokine heteromers prevents neutrophil extravasation in acute lung injury. American Journal of Respiratory and Critical Care Medicine, 185, 628–636. 10.1164/rccm.201108-1533OC 22246174 PMC3326286

[phy214463-bib-0023] Hamacher, J. , Lucas, R. , Lijnen, H. R. , Buschke, S. , Dunant, Y. , Wendel, A. , … Ricou, B. (2002). Tumor necrosis factor‐{alpha} and angiostatin are mediators of endothelial cytotoxicity in bronchoalveolar lavages of patients with acute respiratory distress syndrome. American Journal of Respiratory and Critical Care Medicine, 166, 651. 12204860 10.1164/rccm.2109004

[phy214463-bib-0024] Henriquez, A. R. , Snow, S. J. , Schladweiler, M. C. , Miller, C. N. , Dye, J. A. , Ledbetter, A. D. , … Kodavanti, U. P. (2018). Adrenergic and glucocorticoid receptor antagonists reduce ozone‐induced lung injury and inflammation. Toxicology and Applied Pharmacology, 339, 161–171. 10.1016/j.taap.2017.12.006 29247675 PMC7110430

[phy214463-bib-0025] Ichikawa, A. , Kuba, K. , Morita, M. , Chida, S. , Tezuka, H. , Hara, H. , … Imai, Y. (2013). CXCL10‐CXCR3 enhances the development of neutrophil‐mediated fulminant lung injury of viral and nonviral origin. American Journal of Respiratory and Critical Care Medicine, 187, 65–77. 10.1164/rccm.201203-0508OC 23144331 PMC3927876

[phy214463-bib-0026] Kasahara, D. I. , Kim, H. Y. , Mathews, J. A. , Verbout, N. G. , Williams, A. S. , Wurmbrand, A. P. , … Shore, S. A. (2014). Pivotal role of IL‐6 in the hyperinflammatory responses to subacute ozone in adiponectin‐deficient mice. American Journal of Physiology Lung Cellular and Molecular Physiology, 306, L508–520. 10.1152/ajplung.00235.2013 24381131 PMC3949085

[phy214463-bib-0027] Kawaguchi, N. , Zhang, T. T. , & Nakanishi, T. (2019). Involvement of CXCR4 in normal and abnormal development. Cells, 8(2), 185 10.3390/cells8020185 PMC6406665 30791675

[phy214463-bib-0028] Lehnert, B. E. (1992). Pulmonary and thoracic macrophage subpopulations and clearance of particles from the lung. Environmental Health Perspectives, 97, 17–46. 10.1289/ehp.929717 1396454 PMC1519537

[phy214463-bib-0029] Li, X. , Zhuang, X. , & Qiao, T. (2019). Role of ferroptosis in the process of acute radiation‐induced lung injury in mice. Biochemical and Biophysical Research Communications, 519, 240–245. 10.1016/j.bbrc.2019.08.165 31493867

[phy214463-bib-0030] Looney, M. R. , Thornton, E. E. , Sen, D. , Lamm, W. J. , Glenny, R. W. , & Krummel, M. F. (2011). Stabilized imaging of immune surveillance in the mouse lung. Nature Methods, 8, 91–96. 10.1038/nmeth.1543 21151136 PMC3076005

[phy214463-bib-0031] McDonald, B. , Pittman, K. , Menezes, G. B. , Hirota, S. A. , Slaba, I. , Waterhouse, C. C. , … Kubes, P. (2010). Intravascular danger signals guide neutrophils to sites of sterile inflammation. Science (New York, NY), 330, 362–366. 10.1126/science.1195491 20947763

[phy214463-bib-0032] Michaudel, C. , Fauconnier, L. , Jule, Y. , & Ryffel, B. (2018). Functional and morphological differences of the lung upon acute and chronic ozone exposure in mice. Scientific Reports, 8, 10611 10.1038/s41598-018-28261-9 30006538 PMC6045627

[phy214463-bib-0033] Mubarak, K. K. , Montes‐Worboys, A. , Regev, D. , Nasreen, N. , Mohammed, K. A. , Faruqi, I. , … Antony, V. B. (2012). Parenchymal trafficking of pleural mesothelial cells in idiopathic pulmonary fibrosis. European Respiratory Journal, 39, 133–140. 10.1183/09031936.00141010 21737551

[phy214463-bib-0034] National Toxicology Program . (1994). NTP Toxicology and Carcinogenesis Studies of Ozone (CAS No. 10028‐15‐6) and Ozone/NNK (CAS No. 10028‐15‐6/ 64091‐91‐4) in F344/N Rats and B6C3F1 Mice (Inhalation Studies). National Toxicology Program Technical Report Series, 440, 1–314. 12595923

[phy214463-bib-0035] Nishida, K. , Brune, K. A. , Putcha, N. , Mandke, P. , O'Neal, W. K. , Shade, D. , … Sidhaye, V. K. (2017). Cigarette smoke disrupts monolayer integrity by altering epithelial cell‐cell adhesion and cortical tension. American Journal of Physiology Lung Cellular and Molecular Physiology, 313, L581–l591. 10.1152/ajplung.00074.2017 28642260 PMC5625260

[phy214463-bib-0036] Pan, Z. Z. , Parkyn, L. , Ray, A. , & Ray, P. (2000). Inducible lung‐specific expression of RANTES: Preferential recruitment of neutrophils. American Journal of Physiology Lung Cellular and Molecular Physiology, 279, L658–666. 11000125 10.1152/ajplung.2000.279.4.L658

[phy214463-bib-0037] Roth, S. , Agthe, M. , Eickhoff, S. , Moller, S. , Karsten, C. M. , Borregaard, N. , … Laskay, T. (2015). Secondary necrotic neutrophils release interleukin‐16C and macrophage migration inhibitory factor from stores in the cytosol. Cell Death Discovery, 1, 15056 10.1038/cddiscovery.2015.56 27551482 PMC4979515

[phy214463-bib-0038] Roth, S. , Solbach, W. , & Laskay, T. (2016). IL‐16 and MIF: Messengers beyond neutrophil cell death. Cell Death & Disease, 7, e2049. 26775701 10.1038/cddis.2015.388 PMC4816173

[phy214463-bib-0039] Rush, B. , Wiskar, K. , Fruhstorfer, C. , Celi, L. A. , & Walley, K. R. (2018). The impact of chronic ozone and particulate air pollution on mortality in patients with sepsis across the United States. Journal of Intensive Care Medicine, 885066618804497. 10.1177/0885066618804497 30295138

[phy214463-bib-0040] Scarpelli, E. M. (2003). Physiology of the alveolar surface network. Comparative Biochemistry and Physiology Part A, Molecular & Integrative Physiology, 135, 39–104. 10.1016/S1095-6433(02)00352-5 12727548

[phy214463-bib-0041] Schenkel, A. R. , Chew, T. W. , Chlipala, E. , Harbord, M. W. , & Muller, W. A. (2006). Different susceptibilities of PECAM‐deficient mouse strains to spontaneous idiopathic pneumonitis. Experimental and Molecular Pathology, 81, 23–30. 10.1016/j.yexmp.2005.11.007 16457810 PMC1486780

[phy214463-bib-0042] Schnell, J. L. , & Prather, M. J. (2017). Co‐occurrence of extremes in surface ozone, particulate matter, and temperature over eastern North America. Proceedings of the National Academy of Sciences of the United States of America, 114, 2854–2859. 10.1073/pnas.1614453114 28242682 PMC5358352

[phy214463-bib-0043] Stieb, D. M. , Burnett, R. T. , Beveridge, R. C. , & Brook, J. R. (1996). Association between ozone and asthma emergency department visits in Saint John, New Brunswick. Canada. Environmental Health Perspectives, 104, 1354–1360. 10.1289/ehp.961041354 9118879 PMC1469538

[phy214463-bib-0044] Thanabalasuriar, A. , Neupane, A. S. , Wang, J. , Krummel, M. F. , & Kubes, P. (2016). iNKT cell emigration out of the lung vasculature requires neutrophils and monocyte‐derived dendritic cells in inflammation. Cell Reports, 16, 3260–3272. 10.1016/j.celrep.2016.07.052 27653688 PMC5318399

[phy214463-bib-0045] Thomson, E. M. , Pilon, S. , Guenette, J. , Williams, A. , & Holloway, A. C. (2018). Ozone modifies the metabolic and endocrine response to glucose: Reproduction of effects with the stress hormone corticosterone. Toxicology and Applied Pharmacology, 342, 31–38. 29391239 10.1016/j.taap.2018.01.020

[phy214463-bib-0046] Tighe, R. M. , Li, Z. , Potts, E. N. , Frush, S. , Liu, N. , Gunn, M. D. , … Hollingsworth, J. W. (1950). Ozone inhalation promotes CX3CR1‐dependent maturation of resident lung macrophages that limit oxidative stress and inflammation. Journal of Immunology, 187(4800–4808), 2011 10.4049/jimmunol.1101312 PMC3197861 21930959

[phy214463-bib-0047] Uysal, N. , & Schapira, R. M. (2003). Effects of ozone on lung function and lung diseases. Curr Opin Pulm Med, 9, 144–150. 10.1097/00063198-200303000-00009 12574695

[phy214463-bib-0048] Vanos, J. K. , Cakmak, S. , Kalkstein, L. S. , & Yagouti, A. (2015). Association of weather and air pollution interactions on daily mortality in 12 Canadian cities. Air Quality, Atmosphere & Health, 8, 307–320. 10.1007/s11869-014-0266-7 PMC4449933 26052369

[phy214463-bib-0049] Wang, J. , Hossain, M. , Thanabalasuriar, A. , Gunzer, M. , Meininger, C. , & Kubes, P. (2017). Visualizing the function and fate of neutrophils in sterile injury and repair. Science (New York, NY), 358, 111–116. 10.1126/science.aam9690 28983053

